# Nanoscale chemical mapping of exometabolites at fungal–mineral interfaces

**DOI:** 10.1111/gbi.12504

**Published:** 2022-06-10

**Authors:** Milda Pucetaite, Adam Hitchcock, Martin Obst, Per Persson, Edith C. Hammer

**Affiliations:** ^1^ Department of Biology Lund University Lund Sweden; ^2^ Department of Chemistry and Chemical Biology McMaster University Hamilton Ontario Canada; ^3^ Experimental Biogeochemistry, BayCEER University of Bayreuth Bayreuth Germany; ^4^ Centre for Environmental and Climate Science Lund University Lund Sweden

**Keywords:** Fe(III) reduction, fungal–mineral interactions, NEXAFS, organo–mineral interfaces, soil C sequestration, STXM

## Abstract

Mineral‐associated organic matter is an integral part of soil carbon pool. Biological processes contribute to the formation of such organo‐mineral complexes when soil microbes, and in particular soil fungi, deposit a suite of extracellular metabolic compounds and their necromass on the mineral surfaces. While studied in bulk, micro‐ to nanoscale fungal–mineral interactions remain elusive. Of particular interest are the mutual effects at the interface between the fungal exometabolites and proximal mineral particles. In this work, we have grown saprotrophic and symbiotic fungi in contact with two soil minerals with contrasting properties: quartz and goethite, on top of X‐ray transparent silicon nitride membrane windows and analyzed fungal hyphae by synchrotron‐based scanning transmission X‐ray microscopy in combination with near edge X‐ray fine structure spectroscopy at C(K) and Fe(L) absorption edges. In the resultant chemical maps, we were able to visualize and differentiate organic compounds constituting the fungal cells, their extracellular metabolites, and the exometabolites adsorbing on the minerals. We found that the composition of the exometabolites differed between the fungal functional guilds, particularly, in their sugar to protein ratio and potassium concentration. In samples with quartz and goethite, we observed adsorption of the exometabolic compounds on the mineral surfaces with variations in their chemical composition around the particles. Although we did not observe clear alteration in the exometabolite chemistry upon mineral encounters, we show that fungal–mineral interaction result in reduction of Fe(III) in goethite. This process has been demonstrated for bulk systems, but, to our knowledge, this is the first observation on a single hypha scale offering insight into its underlying biological mechanisms. This demonstrates the link between processes initiated at the single‐cell level to macroscale phenomena. Thus, spatially resolved chemical characterization of the microbial–mineral interfaces is crucial for an increased understanding of overall carbon cycling in soil.

## 1 INTRODUCTION

With global climate change being driven by the increasing concentrations of atmospheric CO_2_, attention is turning to soil as the largest terrestrial carbon sink, where it is stored in a form of soil organic matter (SOM). To understand the principles of this carbon storage capacity, we first need a better mechanistic understanding of the processes responsible for prolonged residence time of SOM compounds in soil.

Some of the most persistent SOM is found associated with soil minerals (Cotrufo et al., 2019; Peng et al., 2017). While such organo‐mineral complexes constitute a dynamic system with a range of interactions taking place at the interface (Kleber et al., 2021), surface‐sorption to minerals has been shown to protect organic compounds from microbial degradation (e.g., Cotrufo et al., 2019; Kaiser, 2003). At the same time, sorbed organic matter (OM) between mineral particles initiates soil aggregation to form complex spatial associations that can provide physical barriers that hide and protect the OM from microbial decomposition, for example, within small pores or within the unconnected interiors of soil aggregates (Lehmann et al., 2007).

Accumulating evidence suggests that a major part of the SOM pool, including mineral associated OM, is of microbial origin. Microbes deposit their extracellular polymeric substances, a suite of smaller metabolic compounds, and, after death, their necromass on the mineral surfaces. Soil fungi in particular, due to their foraging and resource allocation strategies, do not only attract mineral particles that stick to their exometabolite‐covered surface (Costa et al., 2018; Wang et al., 2017), but also administer and transport new carbon compounds into and within soil (See et al., 2022; Vidal et al., 2021).This is especially true for mycorrhizal fungi that get their carbon directly from plants and invest it into biomass and metabolite production which subsequently contribute to the SOM pool (Finlay & Clemmensen, 2017). For instance, arbuscular mycorrhizal fungi (AMF) are globally the most widespread of the symbiotic fungi. With their hyphae extending as deep as 8 m into the subsoil (Sosa‐Hernández et al., 2019), they are able to deliver and deposit organic compounds in the regions of soil not accessible to most decomposers. In addition, AMF produce a specific set of glue‐like glycoproteins which have been linked with increased stability of soil aggregates (Morris et al., 2019; Rillig, 2004; Wright & Upadhyaya, 1998). Ectomycorrhizal (ECM) fungi dominate forest ecosystems and constitute a large part of the total SOM pool there (Finlay & Clemmensen, 2017). They form a vast extraradical mycelium to forage for mineral nutrients (Cairney, 2012) and have even been shown to selectively allocate biomass to different mineral grains of different sizes and surface area (Leake et al., 2008; Smits et al., 2008).

Saprotrophs constitute another large guild of soil fungi. Unlike mycorrhizal fungi, they exclusively obtain carbon by exuding extracellular enzymes to break down OM. Doing so, they alter and distribute carbon compounds in soil. Saprotrophic fungi have also been shown to contribute to increased soil aggregate stability (Demenois et al., 2020) with phylogenetic attribution and hyphal density being the most important factors that positively influence soil aggregation (Lehmann et al., 2020).

The general influence of fungi on soil aggregation and fungal–mineral interactions has been mainly studied in bulk systems. It has been shown that the formation of organo‐mineral complexes is influenced by both the interacting fungal community and soil mineralogy (See et al., 2022 and references therein). Specifically, the sorption potential is determined by the chemistry of the fungal exometabolites (Swenson et al., 2015), which in turn varies in relation to their function: OM decomposition (e.g., various enzymes; Olagoke et al., 2019; Sarkar et al., 1989), mineral weathering (e.g., organic acids; (Adeleke et al., 2017; Jones & Brassington, 1998) or retention of favorable environmental conditions (e.g., secondary metabolites in guttation droplets; (Jennings, 1991; Krain & Siupka, 2021). It also varies depending on the type and crystallinity of the surrounding minerals (Asano et al., 2018; Creamer et al., 2019; Kögel‐Knabner et al., 2008) and the chemical environment (Newcomb et al., 2017).

Fungal metabolites can be retained intracellularly or be released to the extracellular surrounding. Recent research indicates that there is a transition zone between the solid fungal cell‐wall toward completely detached molecules in the matrix of extracellular materials, where many polymeric substances and enzymes can be found loosely connected to the hyphal exterior, comparable to bacterial biofilms (Op De Beeck et al., 2021). Thus, spatial location of the exometabolites around a hypha contributes to determining their level of interaction with the surrounding minerals. Bulk measurements when metabolites are extracted from their spatial context may omit this important information. Therefore, spatially resolved micro‐ to nanoscale studies of organo‐mineral complexes and fungal–mineral interfaces have been receiving increasing attention (Smits et al., 2009).

An extensive review on micro‐ to nanoscale research of microbial–mineral interactions has recently been performed by Finlay et al. (2020)). For instance, atomic force microscopy (AFM), transmission and scanning electron microscopy (TEM, SEM) and light microscopy methods have been used to demonstrate biomechanical effects of fungal–mineral interactions on mineral surfaces, such as formation of nanochannels on silicates (Gazzè et al., 2013; Saccone et al., 2012). In these studies, layers of fungal exometabolites were observed to be deposited on the mineral surfaces, but their chemistry remained unidentified. Bonneville et al. used scanning transmission X‐ray microscopy (STXM) in combination with near‐edge X‐ray fine structure (NEXAFS) spectroscopy at carbon (C), potassium (K), and iron (Fe) absorption edges to show that Fe(II) is oxidized and removed, together with K, from the mineral biotite at the sites of hyphal growth of ECM fungus *Paxillus involutus* (Bonneville et al., 2009, 2016). Similar observations were made by Yu et al., where, in addition to the increase of Fe(II) in the growth medium of the fungus *Trichoderma guizhouense*, formation of biogenic ferrihydrate nanoparticles at the interface between the hyphae and mineral hematite was found (Yu et al., 2020). The authors also used SEM and nanoscale secondary ion mass spectroscopy (nanoSIMS) to confirm the presence of fungal extracellular materials at the sites.

Studies more specifically focused on the chemical composition of the organic materials at the fungal–mineral interfaces were also performed (Keiluweit et al., 2012; Olivelli et al., 2020; Vidal et al., 2018). The interaction between the fungus and the minerals in these studies was taking place in bulk pot or soil systems and the analyses have been carried out on the subsequently extracted hyphae. Furthermore, Keiluweit et al. (2012) used nanoSIMS and STXM to obtain chemical images of the fungal–mineral interfaces, which allowed localization and characterization of the cell–mineral interfaces. Preferential adsorption of microbially altered N containing organic compounds on Fe‐bearing minerals was observed, but, the unambiguous assignment of the origin of these organic depositions remains difficult because the hyphae were recovered from a field site, where many different microbial processes can result in formation of organo‐mineral complexes.

The aim of this work is spatially resolved characterization of the micro‐ to nanoscale chemical interactions between fungal hyphae and soil minerals in a microcosm study. We used synchrotron radiation based STXM‐ NEXAFS microspectroscopy for this purpose. We have grown representative species from each functional guild of fungi—AMF *R. irregularis*, ECM *P. involutus*, and saprotrophic *P. subviscida* and *G. confluence*—in the presence of two common soil minerals—quartz (SiO_2_) and goethite (α‐FeO[OH]). The fungal‐mineral complexes were formed directly on X‐ray transparent silicon nitride (SiN_x_) substrates. This approach allows direct observation of fungal–mineral interfaces without influence of other actors present in natural soils. The selected minerals exhibit very different properties. Goethite, present as agglomerations of needle‐like crystals, has higher surface area than quartz and is also considered to be a reactive mineral that adsorbs high amounts of SOM (Kaiser, 2003). We hypothesized that **(I)** the exometabolites of different fungal species would differ chemically as they have different physiologies and occupy different environmental niches. In particular, we expected the exometabolites of AMF to be more proteinaceous (Hammer & Rillig, 2011) than those of other species. We further hypothesized that **(II)** the different types of exudates, for example, “slime layers” around the hyphae versus guttation, would differ in their composition and concentration of compounds, with guttation droplets being more proteinaceous (Krain & Siupka, 2021) and diluted. In samples with fungal hyphae coming into contact with the mineral particles, we expected species (physiological response) and mineral (chemical selection) specific organo‐mineral complexes to be formed. We hypothesized that **(III)** the presence of minerals would alter the chemical composition of the fungal exometabolites, because environmental triggers as a potential mineral nutrient source may alter the physiology of the hyphae. Secretion of certain fungal secondary metabolites has been shown to be highly mineral‐specific (Li et al., 2021), and Fe is known to be an important element in fungal metabolism. Therefore, we hypothesized that **(IV)** the fungi will respond to the presence of goethite, which will be detected as changes both in the chemical composition of the exometabolites and, potentially, speciation of iron at the goethite surface. Because *P. involutus* has been shown to reduce iron in iron‐bearing minerals and utilize it for nonenzymatic OM degradation (Shah et al., 2020), we further expected that the ECM fungus will demonstrate stronger response to goethite exposure than the other fungi in our study.

In the chemical images obtained through analyses of STXM‐NEXAFS spectral stacks recorded at C(K) and Fe(L) absorption edges, we were able to visualize and differentiate the chemistry of the fungal cell, its extracellular metabolites, and the exometabolites adsorbing on the minerals. We determined both the influence of the adjacent mineral particles on the cell surface and the chemical alterations that the fungal exudation caused in the mineral.

## 2 METHODS

### 2.1 Fungal cultures

Four species of lab‐grown soil fungi—AMF *Rhizophagus irregularis*, ECM *Paxillus involutus*, and saprotrophs *Psilocybe subviscida* and *Gymnopus confluence*—were used in this study. The cultures were maintained in growth media (refer to Supplementary Information for recipes): *R. irregularis* in M growth medium, *P. involutus* in modified Fries medium and the saprotrophs—in malt extract growth medium. In total, we had at least two replicate plates of each fungus per treatment (described below).

### 2.2 Sample preparation

Silicon nitride (SiN_x_) membrane windows (75 nm in thickness and 500 × 500 μm or 750 × 750 μm in size) in square silicon frames (200 μm in thickness and 5 × 5 mm or 2.75 × 2.75 mm in size, depending on the sample holder type used in the particular synchrotron facility) were purchased from Silson Ltd. Quartz (particle size 0.35–3.5 μm) and goethite (powder of single microcrystallites, size not indicated by the producer) were purchased from Sigma‐Aldrich and ThermoFisher Scientific, respectively and used as is. The mineral particles were autoclaved at 120°C for ~20 min and dispersed in 95% ethanol (~10 mg/ml). For the experiment, the SiN_x_ windows were loaded with the minerals by depositing and air drying 6 μl of the particle colloid. Two replicates of each quartz and goethite covered windows as well as one no‐mineral control window were placed directly on the medium in front of the growing fungal colonies (Figure S1).

In case of the AMF fungus, the hyphae had to be directed to grow toward the SiN_x_ windows. For this purpose, a divided petri dish was used, where an inoculum was placed on one side, and a growth medium covered with cellophane film to ensure the growth of hyphae on the surface—on the other. A zigzag pattern in this part of the petri dish was cut out in the medium using a sterile scalpel (see Figure S2) and the SiN_x_ windows were half‐pushed under the cellophane membrane at the edges and tips of the medium cut‐outs.

During growth, the fungi were kept in the dark at 24°C (AMF) or 20°C (all other fungi). The growth was monitored through a stereo microscope and the windows were removed from the plates as soon as at least one hyphal tip reached the middle of the membrane (typically, in 2–3 days for the ECM and saprotrophic fungi). Because the hyphal tips are relatively far away from the growth medium by the time they are on the SiN_x_ membrane (at least 1.25 mm away after having grown over the Si frame around the membrane) and because hyphal tips are built from the vesicles inside of the cell, we did not expect any dragging of the growth medium onto the substrate, and thus its influence to the experiment.

Of note, even with the zigzag cut outs used to direct the single hyphae of the AMF fungus *R. irregularis* toward the SiN_x_ substrates, only one hypha was found grown on a nonmineral control substrate. The hyphae were then cut with a sterile scalpel at the edge of the window before its removal. The removed windows containing hyphae were left to air dry in the sterile hood and then were transferred into gelatin capsule containers for storage and transport. The transfer was done at room temperature in a sterile laminar flow hood.

In the relatively short time that the fungi were grown on the SiN_x_ substrates with or without the minerals, we did not observe any differences in growth rates or patterns between samples. *P. involutus* and *P. subviscida* did not show any changes in growth between mycelium on the medium versus mycelium on the substrate. *G. confluence* had an inconsistent response, with hyphae being scarcer on the SiN_x_ substrate compared to directly on the medium in some cultures. Since SiN_x_ is not considered toxic, we attributed this to an “obstacle effect” where the hyphae redirect instead of growing over the substrate that is 200 μm height. It is difficult to estimate the growth patterns of the *R. irregularis* on the substrates due to the difficulties to direct their growth toward the substrates as described above.

### 2.3 STXM measurements

STXM‐NEXAFS measurements of the samples were performed using the ambient STXM at the SM beamline 10ID‐1, Canadian Light Source (CLS) in Saskatoon, Canada (Kaznatcheev et al., 2007) and I08 beamline, Diamond Light Source in Oxfordshire, UK (https://www.diamond.ac.uk/Instruments/Imaging‐and‐Microscopy/I08.html). The samples on the SiN_x_ membrane windows were mounted on dedicated sample holders: in CLS, the samples were fixed to the trapezoidal aluminum sample holder using double‐sided tape; in Diamond, a sample holder with magnetic lids was used. Mounted samples were placed into the sample chamber of the STXM microscope.

#### 2.3.1 Experimental setup at SM beamline, CLS

**FIGURE 1:**
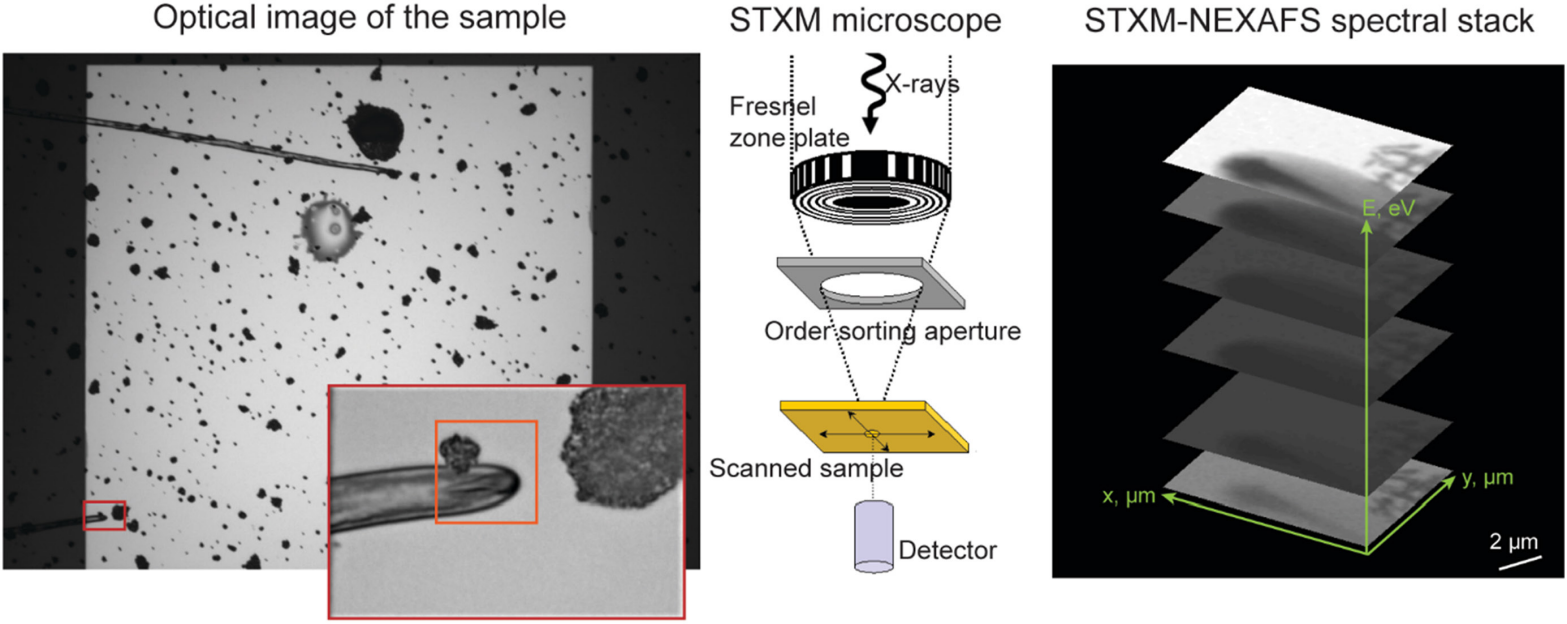
A simplified scheme of synchrotron‐based STXM‐NEXAFS data collection. A ROI is selected in the optical microscopy image of a sample on a SiN_x_ membrane window (left). The window is then placed in/on a sample holder and mounted into the sample chamber of a STXM microscope (middle), where X‐rays from a synchrotron light source are focused onto the sample using a Fresnel zone plate, with the nonfocused part of the beam being blocked by the order sorting aperture. The sample is scanned perpendicular to the beam axis to collect X‐ray transmission information from the ROI. The energy of the beam is then varied and scanning repeated to collect stacks with full spectral information at every point scanned within the ROI image (right)

The ambient STXM sample chamber was evacuated and back‐filled with 16 kPa He gas. Spectral stacks (see Figure 1) were collected using a Fresnel zone plate with an outer zone width of 35 nm, which provided a spatial resolution of ~50 nm at the C absorption edge. The photon energy was monochromated using a plane grating monochromator, and the transmitted X‐rays were detected in single photon counting mode using a phosphor/photomultiplier tube (PMT) detector. Data collection dwell time was typically 1 ms/pixel and the pixel size was typically 50 nm.

#### 2.3.2 Experimental set up at I08 beamline, Diamond Light Source

The microscope sample chamber was evacuated to ~10^−5^ mbar. Spectral stacks were collected using a 480 μm diameter Fresnel zone plate with an outer zone width of 25 nm, which provided a spatial resolution of ~40 nm at the C absorption edge. The photon energy was monochromated by a collimated plane grating monochromator, and the transmitted X‐rays were detected in current mode using a Si photodiode detector. Data collection dwell time was 10 ms/pixel and the pixel size was typically 50 nm.

Both at CLS and Diamond Light Source, NEXAFS spectra at the C(K) absorption edge were collected over the 278–310 eV spectral range. Different spectral regions were sampled as follows: 278–283.4 eV—energy step 0.6 eV, 283.4–292 eV—energy step 0.1 eV, 292–300 eV—energy step 0.3 eV and 300–310 eV—energy step 1 eV. The samples containing goethite mineral particles were also measured at the Fe(L_3,2_) absorption edges in the 705–719 eV spectral range with spectral sampling as follows: 705–709 eV—energy step 0.5 eV, 709–710.5 eV—energy step 0.25 eV, 710.5–715 eV—energy step 0.125 eV, 715–717 eV—energy step 0.25 eV and 717–719 eV—energy step 0.5 eV. Alternatively, images were collected at the absorption pre‐edge (705 eV) and the two main Fe(L_3_) absorption bands at 708.0 and 709.6 eV. Image size was determined by the size of the region of interest (ROI) on each sample. We focused on measuring in the region of the hyphal tips—functionally the most active parts—of first order hyphae (i.e., not recent branches), and the associated mineral particles. Also, we were primarily interested in the chemistry of the fungal extracellular materials and did not perform any detailed analysis of the hyphae themselves as they typically exhibited absorption saturation since the thickness of the hyphae, in the range of 3–5 μm, exceeds the maximum sample thickness of several hundreds of nanometers of carbon compounds (at ~1 g/cm^3^ density) at the C(K) absorption edge spectroscopy. Here we considered that spectra of the hyphae were saturated, and peak intensity dependence on the concentration of absorbing functional groups in the samples was nonlinear, at peak absorbance exceeding values of 1.5, which signals transmission of <3% (see Figure S3).

### 2.4 Electron microscopy measurements

To aid with the analysis, we performed electron microscopy measurements of some of the ROIs within selected samples analyzed by STXM‐NEXAFS. The samples that were measured in CLS (SiN_x_ windows of size 5 × 5 mm) were measured using SEM, while the samples that were measured in Diamond (SiN_x_ windows of size 2.75 × 2.75 mm) were too small for the sample holder of the SEM microscope and were measured using TEM. For SEM measurements, the samples were mounted onto SEM sample stubs, sputter‐coated with gold (Cressington 108 auto, 45 s, 20 mA) and analyzed using a SEM microscope Hitachi SU3500 at 5 kV. TEM measurements were performed using JEOL JEM‐1400 PLUS microscope working at 100 kV. Micrographs were recorded with a JEOL Matataki CMOS camera using *TEM Centre for JEM1400 Plus* software. The electron beam did not penetrate through the fungal cell, but the images provided useful information about thinner exometabolite layer.

### 2.5 STXM data analysis

**FIGURE 2:**
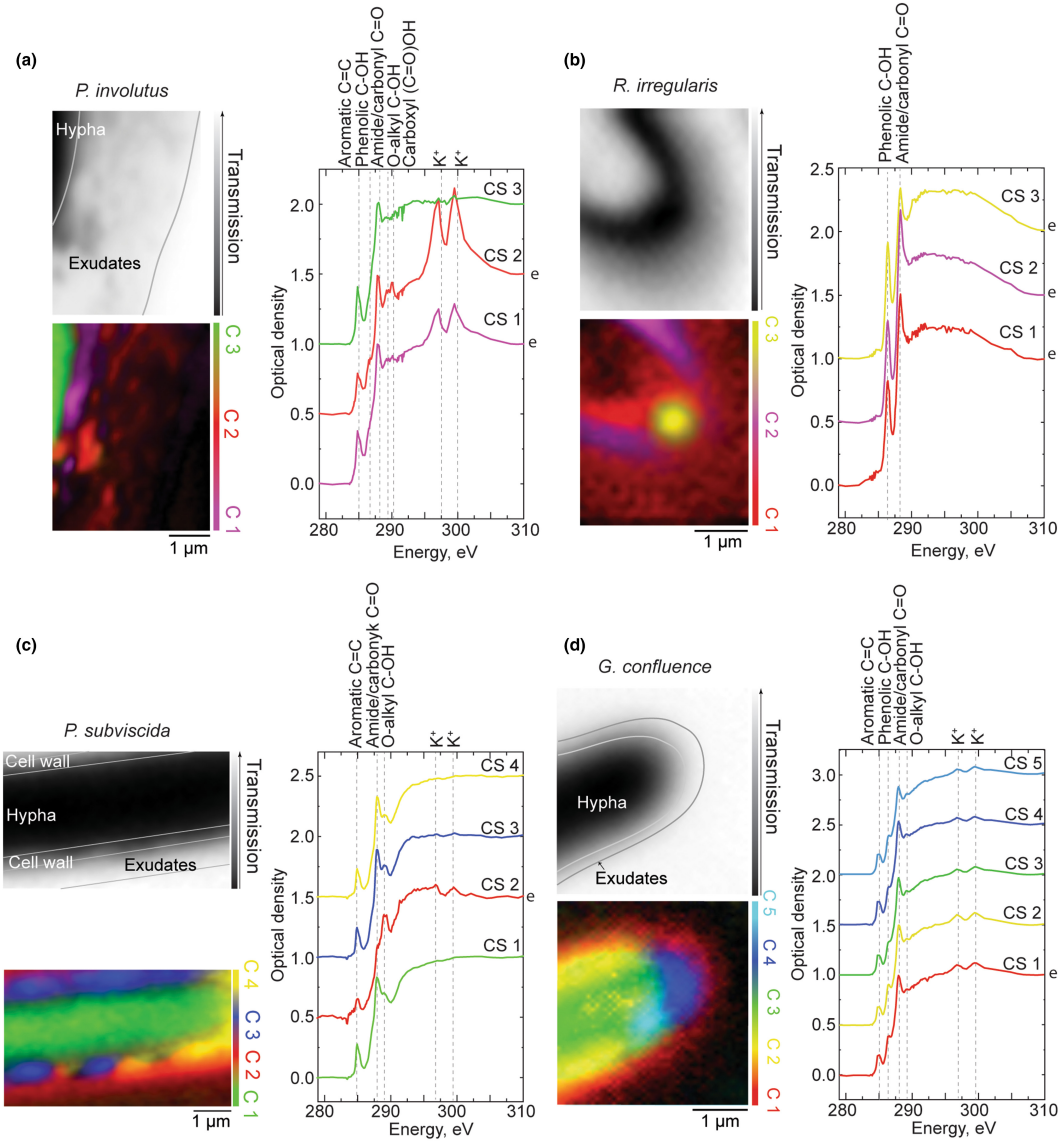
Post‐edge (310 eV) transmission image (top‐left), chemical map (bottom‐left) and corresponding CS (right) of: (a)—ectomycorrhizal *P. involutus* (Diamond Light Source), (b)—AMF *R. irregularis* (Diamond Light Source), (c)—saprotrophic *P. subviscida* (CLS), (d)—saprotrophic *G. confluence* (Diamond Light Source). In the chemical maps, green/yellow/blue colors typically represent the hyphal cell, including the cell‐wall, while red/magenta—the extracellular metabolites. The colors of the CS correspond with the colors of the respective clusters, and the letter “e” on the right of the graph additionally marks the spectra of the exometabolites. The spectra are normalized and shifted in the optical density scale for clarity (0.5 offset for each cluster spectrum except CS1). Refer to Figure S11 for CS in original optical density scale and Figure S5 for microscopy images of the hyphae

**FIGURE 3:**
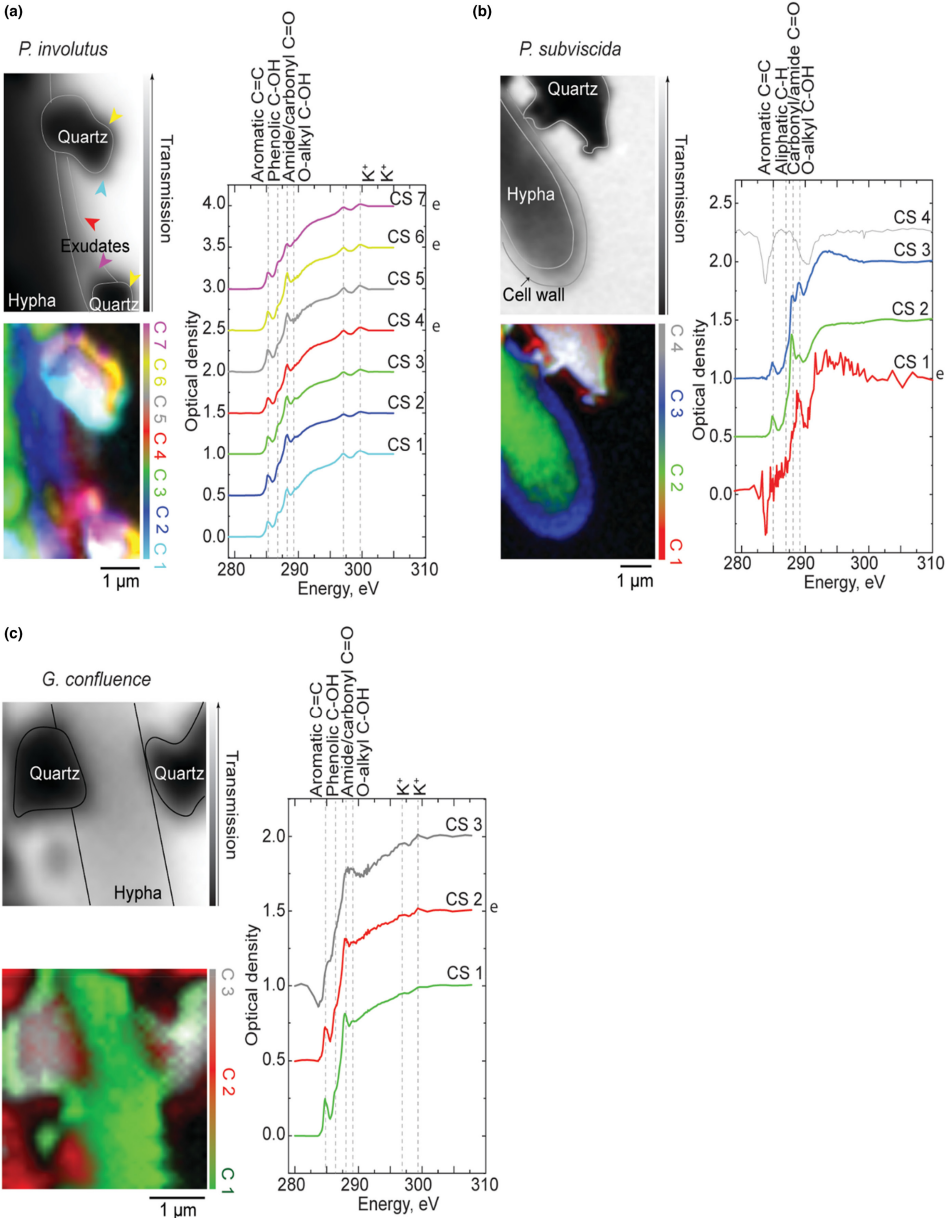
Post‐edge transmission image (top‐left), chemical map (bottom‐left), and corresponding CS (right) of three soil fungal hyphae in contact with quartz particles: (a) ectomycorrhizal *P. involutus* (Diamond Light Source), (b) saprotrophic *P. subviscida* (CLS), (c) saprotrophic *G. confluence* (Diamond Light Source). Note, the post‐edge transmission image in (a) is taken at 306 eV (the last recorded energy value for this sample), while 283 eV pre‐edge transmission image is presented in (c) as it provides more contrast than the respective post‐edge image. In the chemical maps, green color typically represents the hypha, red/magenta—the exometabolites, blue—the cell‐wall and grey—the mineral particles. The colors of the CS correspond with the colors of the respective clusters, and the letter “e” on the right of the graph additionally marks the spectra of the exometabolites. The spectra are normalized and shifted in the optical density scale for clarity (0.5 offset for each cluster spectrum except CS1). Refer to Figure S11 for CS in original optical density scale and Figure 5 for microscopy images of the hyphae

**FIGURE 4:**
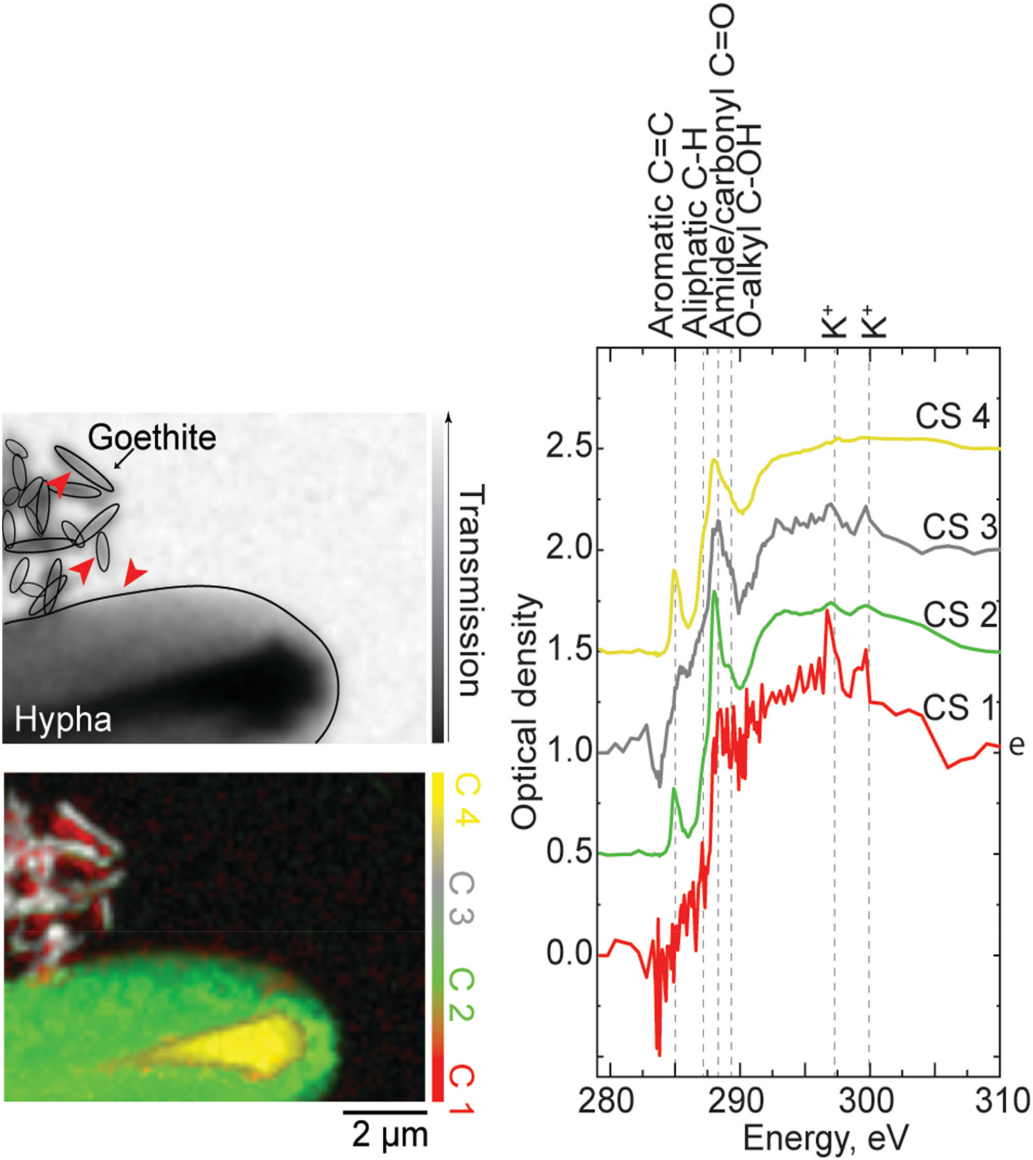
Post‐edge transmission image (top‐left), chemical map (bottom‐left), and corresponding CS (right) of ectomycorrhizal *P. involutus* hypha in contact with goethite particles (CLS): In the chemical map, green/yellow color represents the hypha, red—the exometabolites and grey—the mineral particles. The colors of the CS correspond with the colors of the respective clusters, and the letter “e” on the right of the graph additionally marks the spectra of the exometabolites. The spectra are normalized and shifted in the optical density scale for clarity (0.5 offset for each cluster spectrum except CS1). Refer to Figure S11 for CS in original optical density scale and Figure S5 for microscopy images of the hypha

**FIGURE 5:**
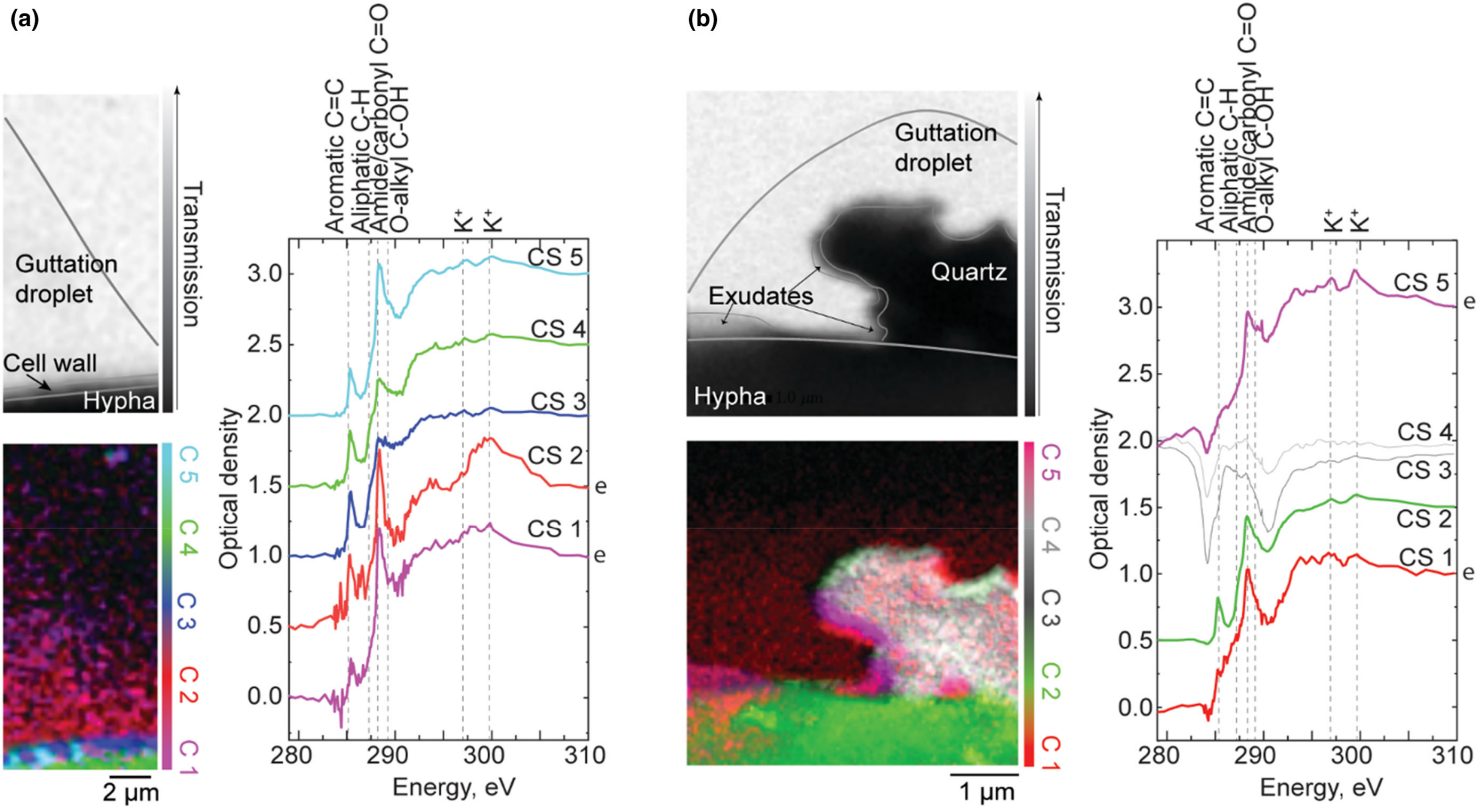
Post‐edge transmission image (top‐left), chemical map (bottom‐left), and corresponding CS (right) of ectomycorrhizal *P. involutus* hyphal tips (CLS) exhibiting guttation on the side of the hypha (a, b) and around the quartz particle (b) that it is in contact with. In the chemical map, green color represents the hypha, red—the exometabolites, blue—the cell‐wall and grey—the mineral particles. The colors of the CS correspond with the colors of the respective clusters, and the letter “e” on the right of the graph additionally marks the spectra of the exometabolites. The spectra are normalized and shifted in the optical density scale for clarity (0.5 offset for each cluster spectrum except CS1; −0.5 offset is applied to CS3 here). Refer to Figure S11 for CS in original optical density scale and Figure S5 for microscopy images of the hyphae

STXM data processing was performed using aXis2000 is written in Interactive Data Language (IDL). It is available free for non‐commercial use from http://unicorn.mcmaster.ca/aXis2000.html and Mantis (Lerotic et al., 2004, 2014) software. For the Diamond Light Source data, the dark signal of the photodiode was first subtracted. Spectral stacks were aligned to compensate for spatial drift. The recorded transmission spectra were converted into optical density, or absorbance ($A=-\ln I/I_{0}$), where *I*_0_ is the signal measured in the regions of the SiN_x_ membrane without the sample. The STXM‐NEXAFS chemical imaging results presented in this work were obtained using multivariate statistical analysis routines described in Lerotic et al. (2004) and embedded in the Mantis software (Lerotic et al., 2014). Briefly, pre‐processed spectral stacks were subjected to principal component analysis (PCA) and the most important components were identified. This was followed by a cluster analysis where the 1st principal component representing the average of the dataset spectra was disregarded in order to reduce the influence of variations in the total thickness within the sample on the analysis results. A power‐law scaling parameter (Lerotic et al., 2004) *ϒ* = 0.3 was used to account for the similar amplitudes of PCA eigenspectra used in the cluster analysis. The number of clusters was varied and selected based on the morphology of the sample and making sure that the average spectrum of each cluster identified is different. Of note, the clusters (C) and the cluster spectra (CS) do not provide information about where separate chemical components are located, but rather show areas that differ in chemical composition more generally. For instance, most CS in all images contained peaks assigned to proteins, but the intensity and position of the peptide peak (Lawrence et al., 2003; Stewart‐Ornstein et al., 2007) varied slightly among the CS relative to other characteristic peaks, such as the strong potassium (L_3_, L_2_) signals at 297.1 and 299.7 eV (Hitchcock et al., 2009; Yoon et al., 2006). The CS presented below in Figures 2, 3, 4, 5 are normalized by subtracting a linear background extrapolated from the pre‐edge region and setting the intensity at 310 eV to 1.

Singular value decomposition (SVD) was then used to calculate pseudo‐thickness images (Lerotic et al., 2004) representing relative contribution of these average CS to each pixel in the image. To clarify, using pure chemical compounds with known X‐ray absorption coefficient values as references in the SVD analysis would yield information about path length of the X‐ray radiation in the sample with known X‐ray absorbance values; in other words—net component thickness of that chemical component. Because here we used the calculated CS in lieu of reference spectra of pure compounds, the SVD result shows relative distributions of the chemical compounds represented by those spectra. These distribution images are referred to as pseudo‐thickness images. For visualization, the pseudo‐thickness image of each component was assigned a color, normalized to maximum intensity and overlaid to create color‐coded pseudo‐thickness images, further referred to as chemical maps, using ImageJ software (Schneider et al., 2012).

Before choosing the multivariate statistical analysis approach described above, we explored a forward fitting (target) analysis using an SVD fit of “hand‐selected” spectra extracted from the sample by using visual inspection of common morphology and spectral features (Hitchcock, 2012; Figure S4). Otherwise, we were also using reference spectra representing the major groups of organic compounds that are expected in biological samples: proteins represented by bovine serum albumin, polysaccharides represented by alginate and xanthan gum, potassium and lipids (Lawrence et al., 2003). We have also performed nonnegative matrix analysis (NNMA) suggested by Lerotic et al. (2014). In the end, we chose to present the results based on cluster analysis as it was less subjective (compared to SVD using hand‐selected spectra or reference spectra, where only limited amount of references could be fitted to the samples of complex chemical composition). The NNMA analysis proved to be unstable; in particular, less intense signals could not be analyzed and identified this way.

Given the complexity of the sample, we did not attempt to identify particular chemical compounds contributing to the C(K) absorption edge spectra, but only specific functional groups indicated by presence of characteristic peaks. Peak energy ranges are those identified by extensive studies of organic compounds relevant to biogeochemistry and soil science (Brandes et al., 2004; Keiluweit et al., 2012; Moffet et al., 2011; Solomon et al., 2009; Zubavichus et al., 2007) and are assigned as follows: nonphenolic aromatic C=C (284–285.5 eV), phenolic C=C/C‐OH or imidazole C=N in histidine (285.8–286.4 eV), aliphatic C‐H/C‐H_2_ (287.1–287.4 eV), amide/carbonyl C=O (287.7–288.6 eV), O‐alkyl C‐OH (289–289.5 eV) and carboxyl COOH (289.5–290 eV). Within the recorded C(K) absorption spectra, there are frequently two intense bands at 297 and 300 eV which are due to L_23_ (2p → 3d) absorption of potassium (K^+^; Hitchcock et al., 2009; Yoon et al., 2006).

The Fe(L) absorption edge NEXAFS spectra were not subjected to the same analysis procedure. Instead, they were normalized to maximum intensity and relative intensities of the two peaks at 708 and 709.6 eV compared. The 708 to 709.6 eV peak ratio is typically used as an estimate of the ratio of Fe(II) to Fe(III) in the sample (Bourdelle et al., 2013). For this particular analysis, we hand‐selected and averaged at least 10 different points in each of the analyzed STXM images, instead of comparing all NEXAFS spectra recorded on the goethite particles. This allowed us to only select points where absorbance was not saturated (transmission was not below 1%–3% and typically higher than 30%) and avoid areas where there was stray light contribution, which would influence the peak ratio values. In the cases where single energy maps were analyzed, the value of transmission was checked at 709.6 eV, where the highest intensity absorption peak is expected. In the case of goethite particles adjacent to fungal hyphae, we selected points that are in direct contact with extracellular materials.

## 3 RESULTS

### 3.1 C(K) absorption edge STXM‐NEXAFS

In the following, we discuss the results from analysis of STXM‐NEXAFS spectral stacks recorded at the C(K) absorption edge of a set of fungal biofilm samples from ECM fungus *P. involutus* and two saprotrophic fungi *P. subviscida* and *G. confluence* grown as no‐mineral controls or in contact with quartz or goethite mineral particles. We have also analyzed one sample of exometabolite print from the AMF *R. irregularis*, as the hypha itself was lost during sample preparation.

#### 3.1.1 Chemistry of exudates of soil fungi

Light microscopy revealed clearly visible exometabolite layers forming around the fungal hyphae, the most pronounced for the ECM fungus *P. involutus* (Figures S5 and S6). We have chosen hyphal tips exhibiting such layers and analyzed them by STXM‐NEXAFS (Figure 2). In the C(K) post‐edge (310 eV) transmission images (e.g., Figure 2a [top‐left]), which provide a contrast based on nonspecific X‐ray absorption by carbon compounds in the sample, the hyphae are generally identified by low transmission values. Regions with intermediate transmission values (typically higher than 30%) at 310 eV correspond to extracellular materials extending around the hyphae, which are also visible in the SEM and TEM microscopy images (Figure S7). In the case of the AMF *R. irregularis* (Figure 2b), the pattern visible in the image is due to the exometabolite print left behind by the fungal hypha that has been lost from the SiN_x_ membrane.

In Figure 2a, the chemical map shows a *P. involutus* hyphal tip (CS 3 in green) and an exudate layer (CS 1 in magenta and CS 2 in red), which extends approx. 2 μm around the tip and is patchy. All CS of *P. involutus* in Figure 2a are dominated by nonphenolic aromatic (further referred to simply as aromatic) and amide/carbonyl peaks. CS 1 and CS 2 of the exudates exhibit additional peaks characteristic to phenolic, aliphatic, O‐alkyl, and carboxyl functional groups. High levels of potassium (K^+^) in the exometabolites are indicated by high intensity K(L_3_, L_2_) absorption edge peaks. Interestingly, the peak characteristic to aromatic (285 eV) compounds is relatively higher in intensity compared to the amide/carbonyl peak (287.9–288.1 eV) in the CS 1 of exometabolites located closer to the hypha, while the O‐alkyl and carboxyl peaks are relatively more intense in CS 2 of exometabolites extending further away from the hypha (see also Table S1 for peak fitting results that highlight the intensity differences).

The CS of the exometabolite print of another mycorrhizal fungus, the AMF *R. irregularis* (Figure 2b), are very different from those of *P. involutus*. They contain only two peaks at 286.3 and 288.3 eV, and lack aromatic, aliphatic, O‐alkyl, carboxyl, or potassium peaks. Having this in mind, we tentatively assign the spectra to amino acid histidine or related compounds, where the two absorption peaks correspond to the imidazole and carboxylate groups (Zubavichus et al., 2007). Additional experiments will have to be carried out to confirm this assignment. Interestingly, the cluster analysis performed on this spectral stack yielded three separate clusters with corresponding CS differentiated by variations in the ratio between the two absorption peaks. Specifically, in the cluster spectrum CS 3 of the circular cluster at the tip of the exometabolite print, the absorption band at 286.3 eV is of relatively higher intensity (Table S1).

The overall observed hyphal apex structure of the two saprotrophic fungi—*P. subviscida* (Figure 2c) and *G. confluence* (Figure 2d)—is resembling the one observed for the ECM fungus *P. involutus* (Figure 2a). The respective hyphae (in green/yellow) are represented by NEXAFS spectra with aromatic and amide/carbonyl peaks. The CS of the *G. confluens* hypha also exhibit a pronounced shoulder at 286.4 eV assigned to phenol functional groups.

With help of SEM (Figure S7) and the chemical maps of the *P. subviscida* hyphal apex (Figure 2c), we interpret CS 1 and CS 4 as the hypha (green and yellow), CS 3—as fungal cell wall (blue) and CS 2—as exometabolite layer (red). An alternating pattern is seen in CS 3 and is related to variations in cell‐wall thickness (see the SEM image in Figure S7). Compared to the cluster spectrum CS 1 of the hypha, the cluster spectrum CS 3 of the cell‐wall has a more pronounced O‐alkyl peak, likely stemming from chitin (Solomon et al., 2009). The exometabolites as well as the cell‐wall contain aromatic, phenolic, amide/carbonyl, and O‐alkyl functional groups. However, the amide/carbonyl to O‐alkyl peak intensity ratio is clearly different in the exometabolites, with the O‐alkyl peak being the dominant. Potassium peaks at 297 and 300 eV are also visible in the CS 2, but do not appear in the other CS, indicating the presence of K^+^ specifically in the extracellular matrix.

Similarly, the clusters in the chemical maps of *G. confluens* apex (Figure 2d) are interpreted as follows: CS 1—exometabolite layer (red), CS 2, CS 3, and CS 4—cell and cell‐wall (yellow, green, and blue), CS 5—an unidentified structure in the hyphal tip (cyan), which is also visible as a circular structure in the TEM image (Figure S7). Only slight variations in the peak ratios (Table S1) and band shapes separate the spectra of different clusters within this sample, making specific spectral signatures difficult to identify. We have iteratively subtracted the cluster spectrum representing the hypha (CS 3) from the other CS in order to identify those signatures characteristic to the organic compounds present in the exometabolite halo and different from the hyphal materials. We used the intensity of the peaks at 285 and 288 eV as guides for determining the subtraction factor. The difference spectra (Figure S8) of exometabolites are dominated by phenol (286.5 eV) and amide/carbonyl (288 eV) peaks, while the spectrum of the circular structure—by a single peak of phenols (286.5 eV).

#### 3.1.2 Exometabolite chemistry in contact with different minerals

Figures 3 and 4 report STXM results from the samples with fungi grown in close proximity of particles of quartz and goethite. In the post‐edge transmission images (Figures 3 and 4, top‐left, pre‐edge transmission at 283 eV provided better contrast for the hypha of *G. confluence*), the quartz particles are identified by their low transmission values, while goethite particles were identified by their needle‐like shape. Some areas on mineral particles displayed the absence of C(K) signal, high absorption (probably significantly saturated), and by negative peaks at 284 and 290 eV (Figures 3b and 5b). The latter are a consequence of rapid changes in 1st and 2nd order light at these energies, due to carbon contamination of the beamline optics (Guttmann & Bittencourt, 2015; Hanhan et al., 2009). On the thinner areas of the particles, the NEXAFS signature of C could be recorded (Figures 3a,c and 4). Such organic depositions were not observed on similar particles far away from the hyphae (Figure S9), implying deposition of fungal exometabolic compounds on their surface. In all the chemical maps, the minerals are represented in grey color.

In the chemical map of *P. involutus* hypha (Figure 3a), four spectral clusters (CS 1, CS 4, CS 6, and CS 7) were assigned to depositions of fungal exometabolites around the hypha (CS 3) and the quartz particles (CS 5), which also yield a C signature. Interestingly, different intensity ratios between aromatic (285 eV), phenolic (286.3 eV) and amide/carbonyl (287.9–288.1 eV) peaks across the clusters indicate variation in chemical composition of the exometabolites depending on their position relative to the hypha and the particles (Table S1). In particular, cluster spectrum CS 7 representing exometabolite deposition located between the hypha and the quartz particle on the lower‐right exhibits a clearly reduced amide/carbonyl peak and relatively more intense peaks characteristic of aromatic and phenolic functional groups compared to CS 4 and CS 6. Furthermore, after analyzing the other CS through subtraction of the CS 3 representing the hypha (Figure S8), we found that CS 1 (organic deposits on the quartz particle side not facing the hypha) exhibits a relatively more intense phenolic peak than CS 6 (organic deposits on the quartz facing the hypha). O‐alkyl peak at 289 eV is only present in CS of the cell‐wall (CS 2), the hypha (CS 3), and the thin exometabolite layer surrounding the hypha (CS 4), with highest intensity in the CS 2, where it is likely stemming from chitin (Solomon et al., 2009). In contrast to the exometabolites in the control sample of *P. involutus* (Figure 2a), the characteristic carboxyl peak expected at 290 eV is not observed.

In the chemical map of *P. subviscida* hypha in contact with quartz particle (Figure 3b), we identify CS 2, CS 3, and CS 4 as the hypha, its cell‐wall, and the quartz mineral particle, respectively. The cluster spectra CS 2 and CS 3 of the hypha and the cell‐wall are consistent with the ones observed in the corresponding control sample (Figure 2c), although CS 3 exhibits a relatively more intense O‐alkyl peak as compared to the amide/carbonyl peak. Cluster CS 1 is identified as a layer of organic substances spread around the mineral and the corresponding spectrum CS 1 is characterized by a single pronounced O‐alkyl peak. Although no distinct exometabolite layer is visible around the hypha itself, we posit that the layer around the particle is caused by fungal activity since the O‐alkyl band at 289 eV was also observed to be the dominant feature in the cluster spectrum of exometabolites of *P. subviscida* hypha in the control sample (Figure 2c). On the other hand, the exometabolite layer around the quartz particle is distinct from the control in lacking the aromatic peak.

In the chemical map of *G. confluence* hypha in contact with quartz mineral particles (Figure 3c), CS 1 is assigned to the hypha, CS 2—to the exometabolites and CS 3—to the mineral. The cluster spectra CS 1 and CS 2 show consistency with the control sample in terms of chemical composition with the most prominent bands being associated with aromatic and amide/carbonyl functional groups, and a shoulder at 286.4 eV characteristic of phenolic functional groups. The latter is of relatively higher intensity in the CS 2 of the exometabolites as well (Table S1). The appearance of the cluster spectrum CS 3 of the mineral suggests there are organic carbon compounds on the surface of the quartz, with the O‐alkyl peak more pronounced than in the spectra CS 1 and CS 2.

We further collected NEXAFS spectral stacks on fungal hyphae in contact with goethite mineral particles. The analyzed hyphal tips of *P. subviscida* and *G. confluence* did not exhibit any evidence of exometabolic materials around the cell or the mineral (Figure S10). This could be due to lack of metabolic activity in the analyzed hyphae.

We did observe organic depositions on the goethite particles in contact with the hyphae of *P. involutus*. In corresponding chemical (Figure 4), they are represented by cluster spectra CS 1 and CS 3. Compared to the other CS, the cluster spectrum CS 1, although noisy, is distinguished by a more pronounced aliphatic peak (287.2 eV) and a blue‐shifted, compared to the other CS, carbonyl/amide (to 288.4 eV) peak. Potassium absorption peaks at 297 and 300 eV are visible in the cluster spectrum as well. While the CS of the *P. involutus* hyphae (CS 2 in this case) are consistent between samples, the very tip visible in this image exhibits saturated absorbance (the absorbance scale of this spectrum reaches values higher than 1.5, indicating saturation, or transmission lower than 3%; Figure S11). This can be explained by the increased thickness resulting from drying of the dense fungal structures, such as Spitzenkörper, at the tip. The thickening is also visible in the SEM image of the tip (Figure S7).

#### 3.1.3 Fungal guttation

Figure 5 presents STXM results for *P. involutus* hyphae exhibiting guttation. The dried guttation droplet is visible on the side of the hypha in no‐mineral control sample as shown in Figure 5 A, both in the post‐edge transmission images and in the corresponding chemical maps. For the hypha in the sample with quartz mineral particles, the thin trace of the guttation droplet is not discernable in the post‐edge transmission image (Figure 5b, the area of the guttation droplet marked based on single‐energy transmission image at 288.2 eV (Figure S12), where the contrast increased due to specific absorption), but is detected by the cluster analysis and represented by cluster spectrum CS 1. The CS of the guttation droplets (CS 1 and CS 2 in Figure 5a and CS 1 in Figure 5b) are dominated by the amide/carbonyl peak at 288.1 eV. The aromatic peak is weak as compared with the other CS identified. In addition to the guttation droplet around the quartz particle (Figure 5b), a layer of organic substances as represented by C 5 are found adhering to its surface. The corresponding CS 5 is also dominated by the amide/carbonyl peak, but is distinguished by the presence of a shoulder, assigned to O‐alkyl functional group, and a weak aromatic peak. These chemical differences between the guttation droplet and the exometabolite layer suggest that they are secreted separately and, in this case, particularly the exometabolites contribute to the adsorbed substances visible around the quartz mineral particle.

Other than the guttation, no exometabolite layer is visible around the hypha in the control sample (Figure 5a). The clusters CS 3 and CS 5 are assigned to be the cell‐wall after comparing with the corresponding SEM image of the tip (Figure S7). The alternating pattern seen between these two clusters is due to a different thickness effect picked‐up by the cluster analysis. The cluster spectra CS 3 and CS 5 exhibit relatively more intense aromatic, aliphatic, and O‐alkyl peaks, compared to the other CS (Table S1). The O‐alkyl peak has been observed to be relatively more intense in the cell‐wall spectra of all fungi, including *P. involutus* in Figure 3a, likely due to the presence of chitin (Solomon et al., 2009). The CS of hyphae—CS 4 in Figure 5a and CS 2 in Figure 5b—are consistent with corresponding spectra of *P. involutus* hyphae in other, including control, samples.

### 3.2 Fe(L_3_ ) absorption edge STXM‐NEXAFS

**FIGURE 6:**
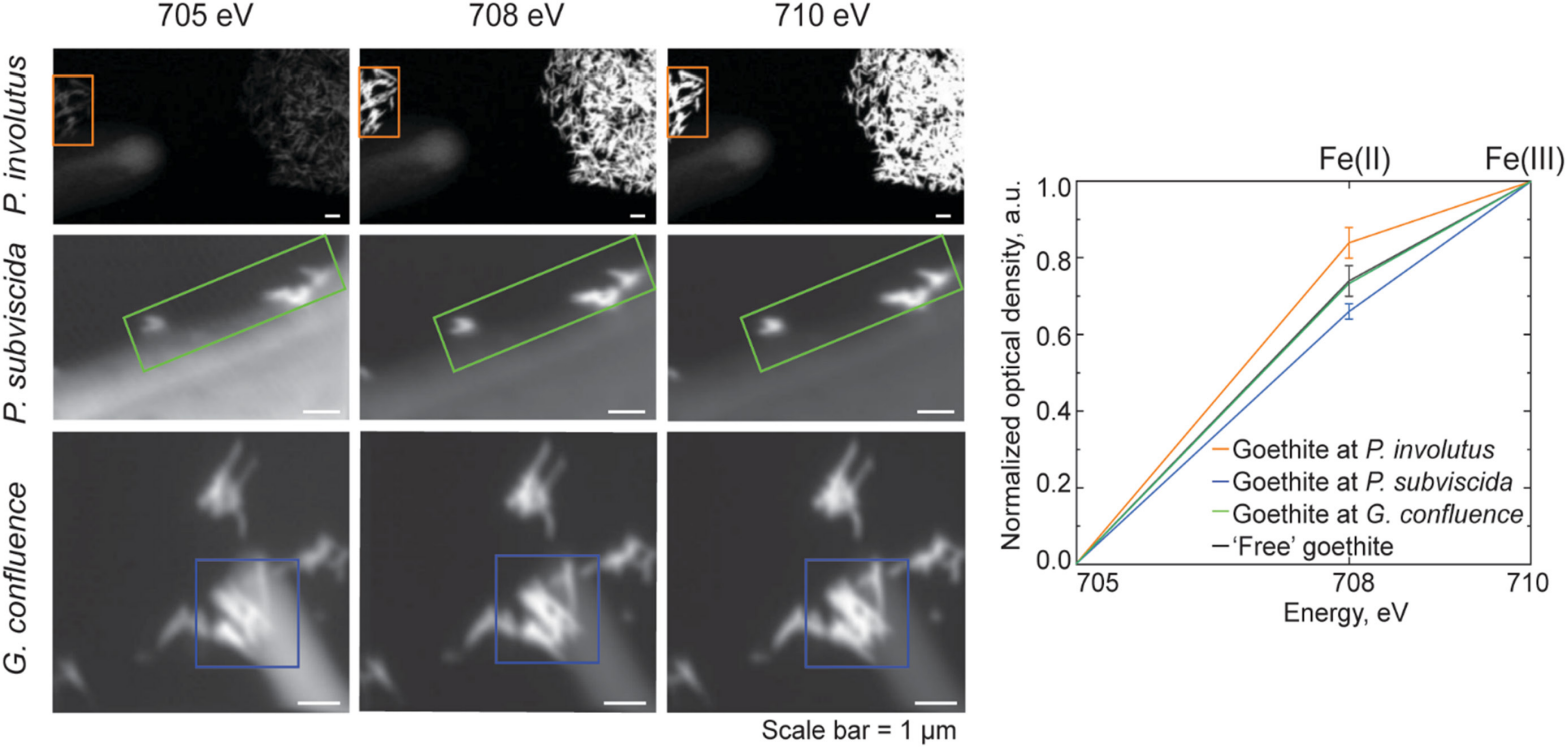
Goethite mineral particles in contact with *P. involutus*, *P. subviscida*, and *G. confluence* fungal hyphae (images on the left) and normalized NEXAFS absorption intensity graph (on the right) showing changes in relative intensity of 708 eV peak corresponding with changes in relative content of Fe(II) in respect to Fe(III) in the particles. The STXM images here are provided as an example, and the rectangles mark the areas where point spectra were selected for further analysis from (see Materials and Methods). Numerical values of peak intensities in replicate images as well as statistical analysis reports are provided in Table S2

Since it is known that fungi have the capacity to reduce iron in minerals (Ottow & Von Klopotek, 1969; Shah et al., 2020), we specifically recorded STXM‐NEXAFS images at the Fe(L_3_) absorption edge of samples containing goethite particles. The relative intensity of the two peaks at 708 and 709.6 eV in Fe(L_3_) absorption spectra can be indicative of iron speciation: higher intensities of the 708 eV band relative to the 709.6 eV band typically suggest higher levels of Fe(II) than Fe(III) in the sample (Bourdelle et al., 2013). The optical density values (Figure 6), which are normalized to 1.0 at 709.6 eV, show changes in the intensity of the 708 eV peak relative to the 709.6 eV peak when the signal is recorded on goethite particles alone versus in contact with *P. involutus*, *P. subviscida*, and *G. confluence* hyphae. The Fe(II)/Fe(III) ratio is the highest in the goethite mineral particles in contact with *P. involutus* hyphae and significantly (*p* < .01, *n* = 3) higher than in the control particles. In contrast, we could not find an indication of the saprotrophic fungi affecting Fe speciation at this level of replication (*n* = 2). This could also be explained by the fact that we did not observe exometabolites around the analyzed hyphae of these fungal species.

## 4 DISCUSSION AND FUTURE PERSPECTIVES

Fungi exude a variety of chemical compounds that serve different functions, such as to help them to retain moisture, acquire nutrients, sense the environment, send, and receive chemical communication signals or form symbiotic interactions (Op De Beeck et al., 2021). Generally, they can be categorized as primary exometabolites—constitutional compounds that are always exuded during growth—or as secondary, induced exometabolites that depend on environmental or biotic stimuli (Karlovsky, 2008). The former are necessary for survival of the fungus, orientation of hyphae, etc., and the latter are essential for exploiting nutrient sources, reacting to adverse abiotic or biotic environments, such as to toxins or competition, respectively. Thousands of different chemical compounds have been identified in fungal exometabolites extracted from bulk cultures (Li et al., 2021). Here, we used STXM‐NEXAFS to explore chemical composition and spatial distribution of the exometabolites around apexes of single hyphae with nanoscale resolution. As expected for cytoplasmic materials comprising the hyphae, features observed in the corresponding CS are characteristic to proteins (Stewart‐Ornstein et al., 2007). In the layers of exometabolites and guttation droplets, we were able to identify groups of chemical compounds—at the level of chemical specificity allowed by NEXAFS spectra—such as proteins, polysaccharides, or phenolic compounds. We observed exometabolite layers extending up to 2 μm around the hyphae and in some cases forming a patchy pattern in terms of thickness (Figure 2a). Similar structures have been observed by Gazze et al. through AFM of *P. involutus* hyphae grown on phyllosilicate sheets (Gazzè et al., 2013). They interpreted the patchy pattern as an indication of active involvement of the hyphal apex in the exometabolite layer formation. Interestingly, we also found that the chemical composition of the exometabolites slightly varies across their spatial extent (Figure 2). The highly regular patterns of these variations identified by cluster analysis (refer to the chemical map of AMF *R. irregularis* in Figure 2b) can be formed by segregation of compounds during drying of the liquid layer of exometabolites (Pathak et al., 2020; Tarasevich & Pravoslavnova, 2007). However, we also repeatedly observed clusters representing fungal cell wall forming oscillating patterns (Figures 2c and 5a), with alternating thicker and thinner layers, and with associated oscillating patterns of K^+^ concentration (Figure 2c). The underlying reason for these patterns might potentially be physiological due to differences in the structure of the wall at different parts of the hyphae, such as nonrandomly distributed K‐channels found in *Neuraspora crassa* or *Saprolegnia ferax* (Aslanidi et al., 2001; Lew, 1998). Similarly, patterns in the exometabolites could be the result of segregated exudation channels/pumps secreting different compounds into extracellular space. We have no direct evidence for morphological causes at this stage.

Fungal exudation can generally differ among taxonomic/functional groups (Frisvad et al., 2008) and our results supports this, although more extensive sampling and more replicates are necessary for further confirmation. The exometabolites of all the fungal species analyzed in this work contain peptides as evidenced by the amide/carbonyl peak in the NEXAFS spectra. However, the overall exometabolite chemistry differed among them, as evidenced by position shifts and intensity of the amide/carbonyl peak relative to other peaks in the NEXAFS spectra. For instance, the exometabolites of *P. subviscida* are dominated by polysaccharide compounds, with significantly higher O‐alkyl to amide/carbonyl peak ratio than observed in other samples (Figures 2 and 3). The most different chemical features, as expected, were found in the exometabolites of AMF *R. irregularis*. We tentatively assigned the spectral features in Figure 2 B to amino acid histidine. Up‐regulation of genes encoding secretion of, among others, histidine‐rich proteins in AMF fungi has been associated with facilitation of establishing symbiosis with a host plant (Kamel et al., 2017; Prasad Singh et al., 2019), which could explain the spectral signature of the amino acid in the NEXAFS spectra. However, more experiments are necessary to confirm our assignment.

*R. irregularis* is also the only species where the exometabolites did not exhibit any spectral signatures of K^+^. On the contrary, the exometabolite chemistry of *P. involutus* yields a strong signal at the K(L) absorption edges which indicates high amounts of K^+^ ions present (Figure 2a). In fungal cells, K^+^ ions are secreted through ion pumps in cellular membrane to retain ionic homeostasis between intra‐ and intercellular environment (Rodríguez‐Navarro, 2000). At this point, it is not clear why exometabolites of the AMF *R. irregularis* hyphal tip would lack them entirely.

The *P. involutus* hyphal exometabolites in the no‐mineral control sample likely contains organic acids, as evidenced by presence of the peak at 290.1 eV assigned to carboxyl compounds. However, the exometabolite composition among the *P. involutus* hyphae analyzed here was not consistent and in several cases the hyphae did not have the exometabolite layer at all (Figure S13). This, as has been shown by Op De Beeck et al. (2020), can likely be attributed to different functional activity of individual hyphae within the mycelium.

Hyphae of fungus *P. involutus* were also producing guttation droplets—active exudation of water and water‐soluble compounds (Figure 5). They were determined to be mostly proteinaceous in nature. However, the NEXAFS signal from the dried guttation areas was weaker than the signal from the areas of the exometabolite layers, indicating that the concentration of the proteins within the original aqueous droplets was probably low. Guttation in fungi is known, but largely understudied. Some studies have shown that the guttation droplets contain enzymes and can perform the function of OM degradation (Gareis & Gottschalk, 2014). Meanwhile, Jennings (1991) has postulated that guttation acts as a water reservoir for fungi to be able to maintain favorable water potential and grow away from their nutrient source, which might be the case in our system with them growing on the dry SiN_x_ surface. On the other hand, they might also be exuded as a response to environmental triggers, such as presence of toxins (see citing articles of Jennings, 1991). Generally, we did not observe any other signs of toxicity of SiN_x_ to the fungus *P. involutus*.

When grown in contact with minerals, fungi deposit their exometabolites on their surface and thus contribute to mineral‐associated organic matter pool that is key for C storage in soils (Kleber et al., 2015). Our observations of organic (C(K)) signal on the mineral particles confirm this. Given the different functions associated with fungal–mineral interactions (nutrient acquisition, physical protection, etc.), we hypothesized that the fungal response to coming into contact with different minerals will result in a change in the chemical composition of their exometabolites. For instance, it has been demonstrated that production of certain secondary metabolites in the fungus *Talaromyces flavus* is highly mineral‐specific (Li et al., 2021). We expected this change to be greater for goethite, since Fe is known to be an important element for fungal metabolism, particularly for *P. involutus*, which has been shown to utilize Fe for nonenzymatic OM degradation (Shah et al., 2020). Indeed, we have observed variation in the intensity of the aromatic peak (285 eV) in the NEXAFS spectra of exometabolites of the fungal hyphae growing in contact with the mineral particles (Figures 3 and 4) as compared to the ones in the no‐mineral control samples. However, no significant changes in exometabolite chemistry was observed among hyphae growing in contact with quartz and goethite. This could be due to the relatively brief contact time between the minerals on the SiN_x_ substrates and the hyphae in our experiments. Therefore, experiments where the hyphae are exposed to the minerals for a longer period of time should be carried out in the future. On the other hand, the changes could be too small to discern given limited chemical resolution of NEXAFS spectroscopy as well as low concentration of mineral‐specific secondary metabolites.

It has been previously shown by STXM‐NEXAFS that small organo‐mineral associations extracted from Andisol soils exhibited spectral peaks of aromatic, phenolic and amide groups which were assigned to microbially‐altered organic compounds (Asano et al., 2018). Here, we also show that those same functional groups are present in the fungal exometabolites, and when grown in contact with mineral particles, they wet the particles to adsorb on their surface.

Although at the level of chemical resolution provided by STXM‐NEXAFS, we did not observe clear alteration in the exometabolite chemistry upon different mineral encounters, here we show that fungal–mineral interaction results in reduction of iron in goethite (Figure 6)—a process that has been demonstrated before for bulk systems (Ottow & Von Klopotek, 1969; Shah et al., 2020), but for the first time observed on a single hypha scale. Specifically, ECM fungus *P. involutus* has been shown to act on the iron in goethite particles up to a 2 μm away from the hyphal cell, which is the extension of exometabolites layers observed here. This is in line with evidence provided in earlier studies, where it has been shown to employ nonenzymatic Fenton's chemistry based pathways for OM decomposition (Shah et al., 2020). In our experimental design, the analyzed fungal hyphae were growing on culturing plates before transitioning to the smooth and nutrient‐free substrates of the SiN_x_ windows with randomly distributed goethite particles. Fe reduction consistently taking place in such systems suggests either constitutive expression of the reducing functions by the fungi or their quick physiological reaction to the encounter with the particles. Interestingly, we did not observe any obvious NEXAFS peaks characteristic of compounds typically associated with iron reduction, such as siderophores (Thieme et al., 2013; Winkelmann, 2007) or quinones (Lyngsie et al., 2018; Solomon et al., 2009), in the hyphal exometabolites. This could be due to their low concentration relative to other exometabolites, or other compounds being responsible for the process.

Several limitations of our study on nanoscale chemistry of fungal exometabolites and organo–mineral interfaces formed through fungal–mineral interactions could be solved in the future. First, in order to better identify differences in the exometabolite chemistry and subsequently function among fungi from different guilds, expanding the set of species and replicates in the analysis is an important step. Analyzing more hyphal tips within a sample would also allow further addressing the issue of hyphal phenotypical heterogeneity where some hyphae are actively producing exometabolites while others are not. While STXM‐NEXAFS provides excellent spatial resolution for nanoscale analysis, combining it with other methods such as X‐ray fluorescence microscopy (XRF) or nanoscale infrared microspectroscopy could provide more detailed information about the chemistry of the exometabolites. Finally, the next step to study fungal iron reduction in goethite should be setting up experimental systems including an OM substrate. This could contribute to explaining the role of iron and iron‐bearing minerals in fungal SOM decomposition.

## 5 CONCLUSIONS

In this work we show that, at the level of chemical resolution provided by STXM‐NEXAFS, fungal exometabolites differ between fungal functional guilds, particularly, in their sugar to protein ratio and potassium concentration. The guttation droplets produced by *P. involutus* were, furthermore, different as compared to the exometabolites layers produced by the fungus, with the nonaromatic peptides dominating the NEXAFS spectra. In samples with quartz and goethite particles, we observed wetting behavior of the exometabolites, where they extend up to several micrometers away from the hyphal tip and likely constitute the “glue” for fungal soil aggregation that can clearly be observed at larger scales. Contrary to our expectation, we did not observe a clear alteration in the exometabolite chemistry upon different mineral encounters in any of the analyzed fungi; further research is needed to address this issue.

We did demonstrate that iron reduction in goethite takes place at the hyphal–mineral interfaces of ECM fungus *P. involutus*, even without an organic substrate that would trigger iron‐chemistry driven decomposition pathways. This demonstrates the contribution of the processes initiated at single‐cell level to the corresponding macroscale observations that have been made in previous studies. It also highlights the relevance of analytical approaches such as STXM to provide highly spatially resolved chemical characterization of the microbial–mineral interfaces for increased understanding of the processes taking place in soil at the scale of their origin, which will further enable better description of the OM and, more generally, carbon cycling at ecosystem scale.

## CONFLICT OF INTEREST

The authors declare no conflict of interest.

## Supporting information


Appendix S1

## Data Availability

The data that support the findings of this study are openly available in Mendeley Data at https://data.mendeley.com/, reference number DOI: 10.17632/55yj7ypsmm.1.
