# Supplementary material for: Nanoscale chemical mapping of exometabolites at fungal–mineral interfaces
Source: Geobiology. 2022 Jun 10;20(5):650–66. doi: 10.1111/gbi.12504 (PMC9546123; doi:10.1111/gbi.12504)
Supplement: Supplementary file 1 — Appendix S1 [file GBI-20-650-s001.doc]

**Supplementary Information**

**Nanoscale chemical mapping of exometabolites at fungal-mineral interfaces**

Milda Pucetaite^1*^, Adam Hitchcock^2^, Martin Obst^3^, Per Persson^4^, Edith C. Hammer^1^

^1^Department of Biology, Lund University, Lund, Sweden

^2^Department of Chemistry & Chemical Biology, McMaster University, Hamilton, ON, Canada

^3^Experimental Biogeochemistry, BayCEER, University of Bayreuth, Bayreuth, Germany.

^4^Centre for Environmental and Climate Science, Lund University, Lund, Sweden

*Corresponding author: [milda.pucetaite@gmail.com](mailto:milda.pucetaite@gmail.com); [milda.pucetaite@biol.lu.se](mailto:milda.pucetaite@biol.lu.se)

**Growth media descriptions**

*For the saprotrophic fungi P. subviscida and G. confluence:*

**Malt extract growth medium (ME).** Malt extract 2% containing 10 ml/l vitamin stock solution and 1ml/l trace metal solution

**Trace metal solution.** 1 g ZnSO_4_∙7H_2_O and 0.5 g CuSO_4_∙5H_2_O disolved in 100 ml milliQ water

**Vitamin stock solution.** Per liter of milliQ water: 1 g inositol; 0.01 g thiamine-HCl; 0.0025 g biotin; 0.01 g pyrodoxine; 0.01 g riboflavine; 0.01 g nicotinamide; 0.01 g p-aminobenzoic acid; 0.01 g Ca-panthotenate

*For the ECM fungus P. involutus:*

**Modified Fries growth medium.** Per liter of milliQ water: 10 ml of each stock solutions 1, 2, and 3; 10 ml vitamin stock solution (see above); 0.2 g NH_4_Cl; 0.02 g FeCl_3_∙6H_2_O; 6 g D-glucose, pH adjusted to 4.8

**Stock solution 1.** Per liter of milliQ water: 10 g MgSO_4_∙7H_2_O; 2 g NaCl; 10 g KCl; 1.5 g H_3_BO_3_; 0.575 g ZnSO_4_∙7H_2_O; 0.125 g CuSO_4_∙5H_2_O; 0.85 g MnSO_4_∙H2O; 0.02 g (NH_4_)_6_Mo_7_O_24_∙4H_2_O and 2.72 ml HCl (37 %)

**Stock solution 2.** Per liter of milliQ water: 3 g KH_2_PO_4_ and 2.72 ml HCl (37 %)

**Stock solution 3.** Per liter of milliQ water: 2.6 g CaCl_2_∙2H_2_O and 2.72 ml HCl (37 %)

*For the AMF R. irregularis:*

**M-medium (**Ref. Bécard &Fortin 1988 NPhyt 108). Per liter of milliQ water: 25 ml of stock solution 1, 5 ml each of stock solutions 2 and 3, 1 ml each of stock solutions 4 and 5, 10 ml of stock solution 6, 10 g sucrose, pH adjusted to 5.5

**Stock solution 1.** Per liter of milliQ water: 29.24 g MgSO_4_∙7H_2_O; 3.2 g KNO_3_; 2.6 g KCl

**Stock solution 2.** Per 100ml of milliQ water: 96 mg KH_2_PO_4_

**Stock solution 3.** Per 100 ml of milliQ water: 5.76 g Ca(NO_3_)_2_∙4H_2_O

**Stock solution 4.** Per 100 ml of milliQ water: 800 mg Na-Fe-EDTA

**Stock solution 5.** Per 100ml of milliQ water: 75 mg KI; 600 mg MnCl_2_∙4H_2_O; 265 mg ZnSO_4_∙7H_2_O; 150 mg H_3_BO_3_; 13 mg CuSO_4_∙5H_2_O; 1 ml Solution A

**Solution A.** Per 100ml of milliQ water: 24 mg Na_2_MoO_4_

**Mother solution 6.** Per 200ml of milliQ water: 1 ml Solution B; 60 mg glycin; 1000 mg myo-inositol

**Solution B.** Per 10ml of milliQ water: 20 mg thiamin-HCl; 20 mg pyridoxin-HCl; 50 mg nicotine acid

If stored, stock solutions 1-5 are stored in refrigerator, stock solution 6 in the freezer (-20°C) and stock solution 4 has to be covered to protect from light.

**Agar concentrations.** For all except M growth medium we used 1,5 % agar. For M medium 3 g/l of phytagel was used instead


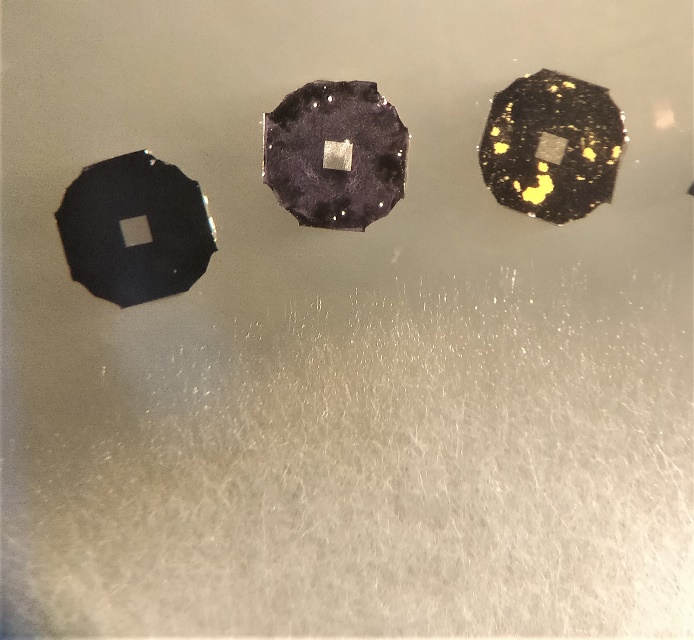


**SI Figure 1.** Stereomicroscopy image of 2.75×2.75 mm frame size SiN_x_ membrane windows in front of a fungal colony (*P. subviscida*) in a Petri dish. Samples from the left: control, quartz, goethite


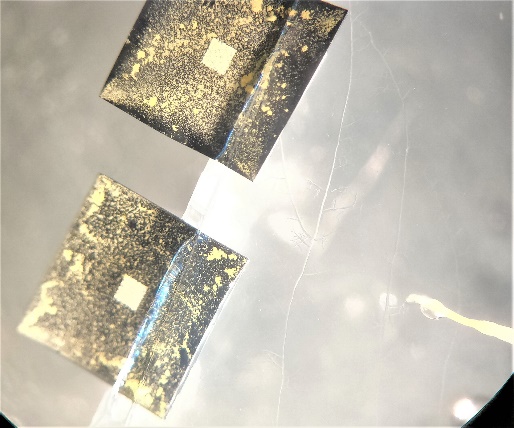

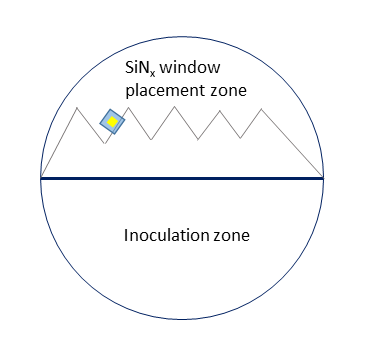


**SI Figure 2.** AMF growth example with cut-out patterns in medium to direct hyphal growth towards the windows


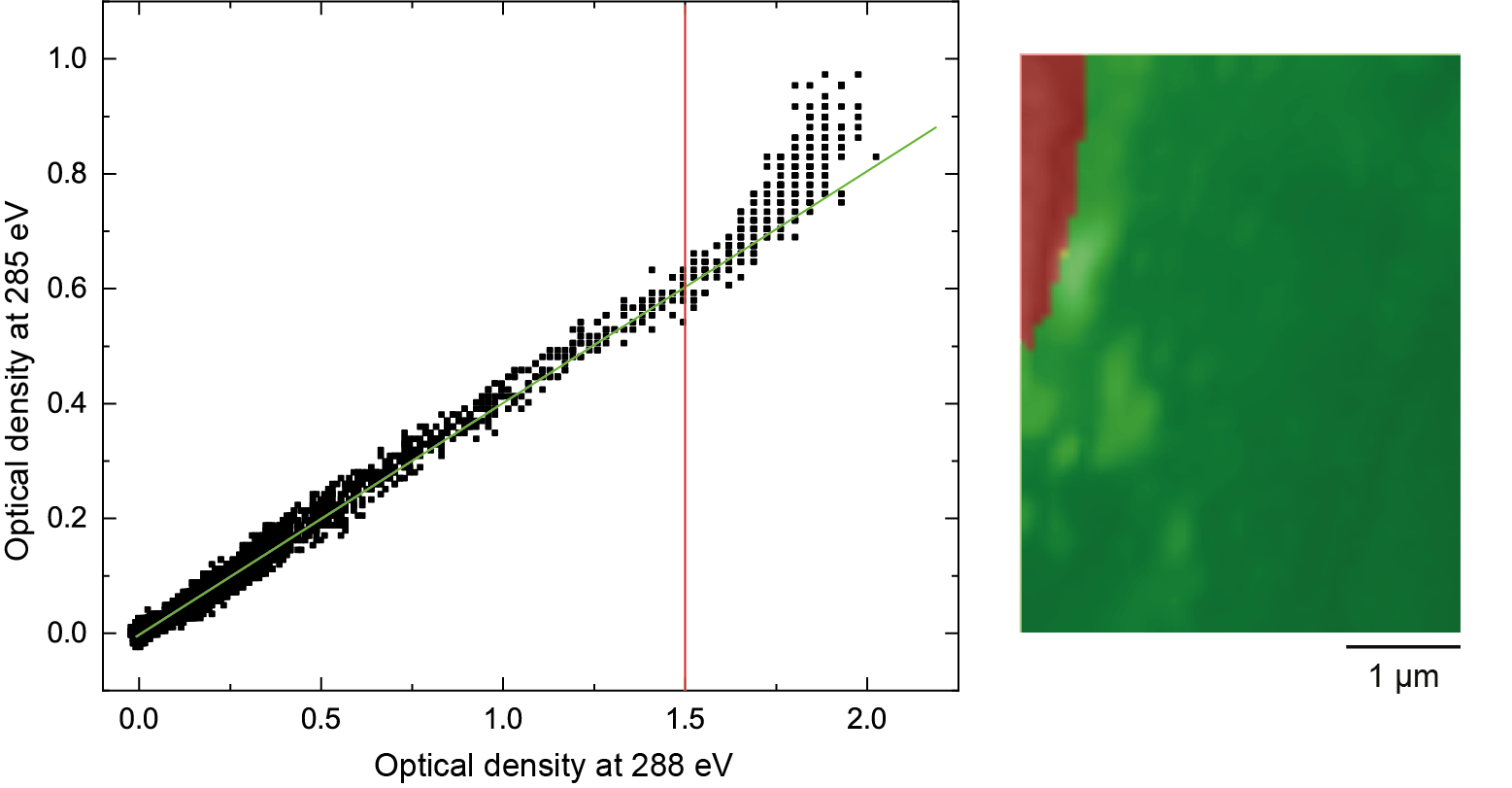


A

B


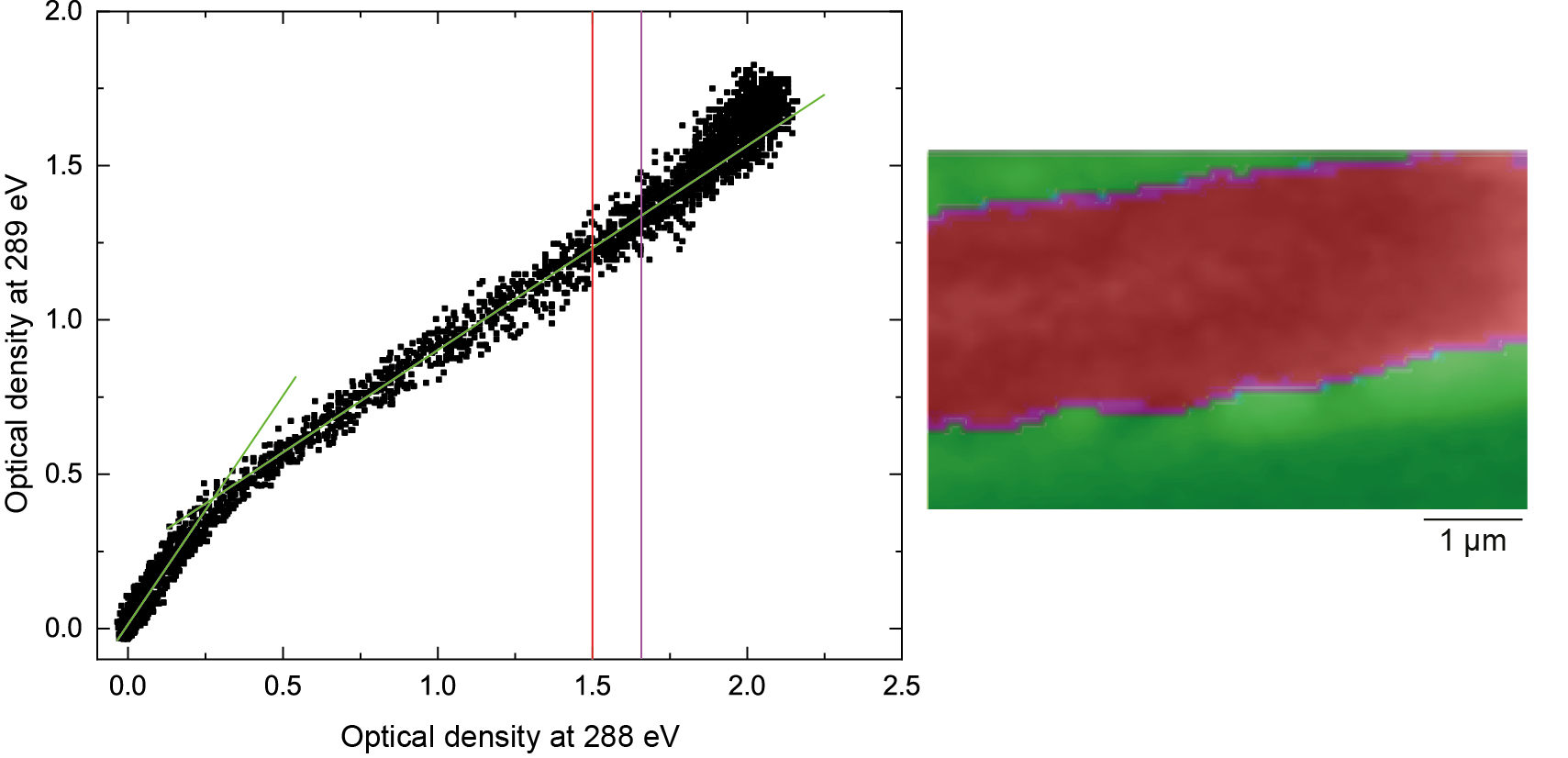


C


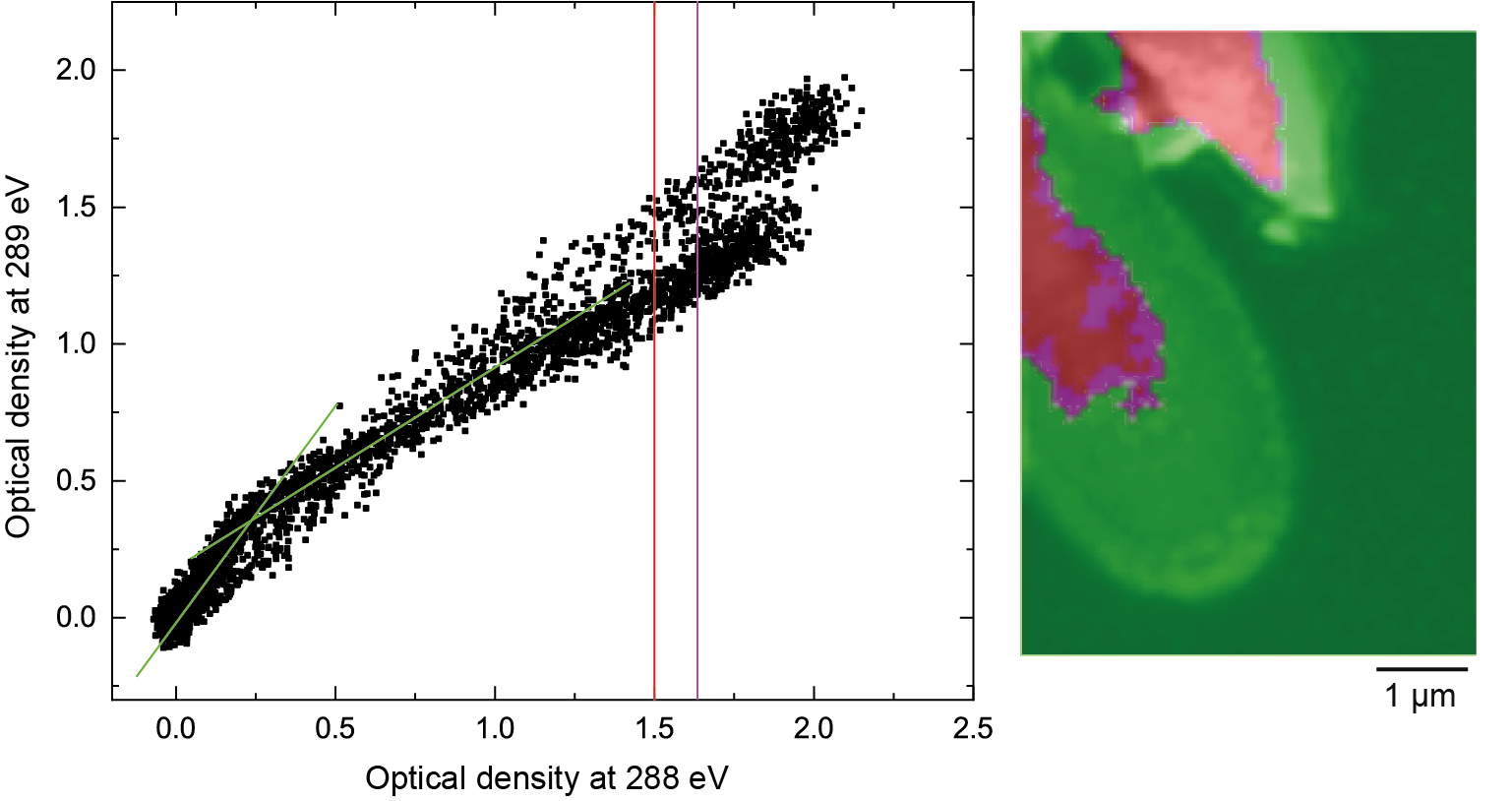


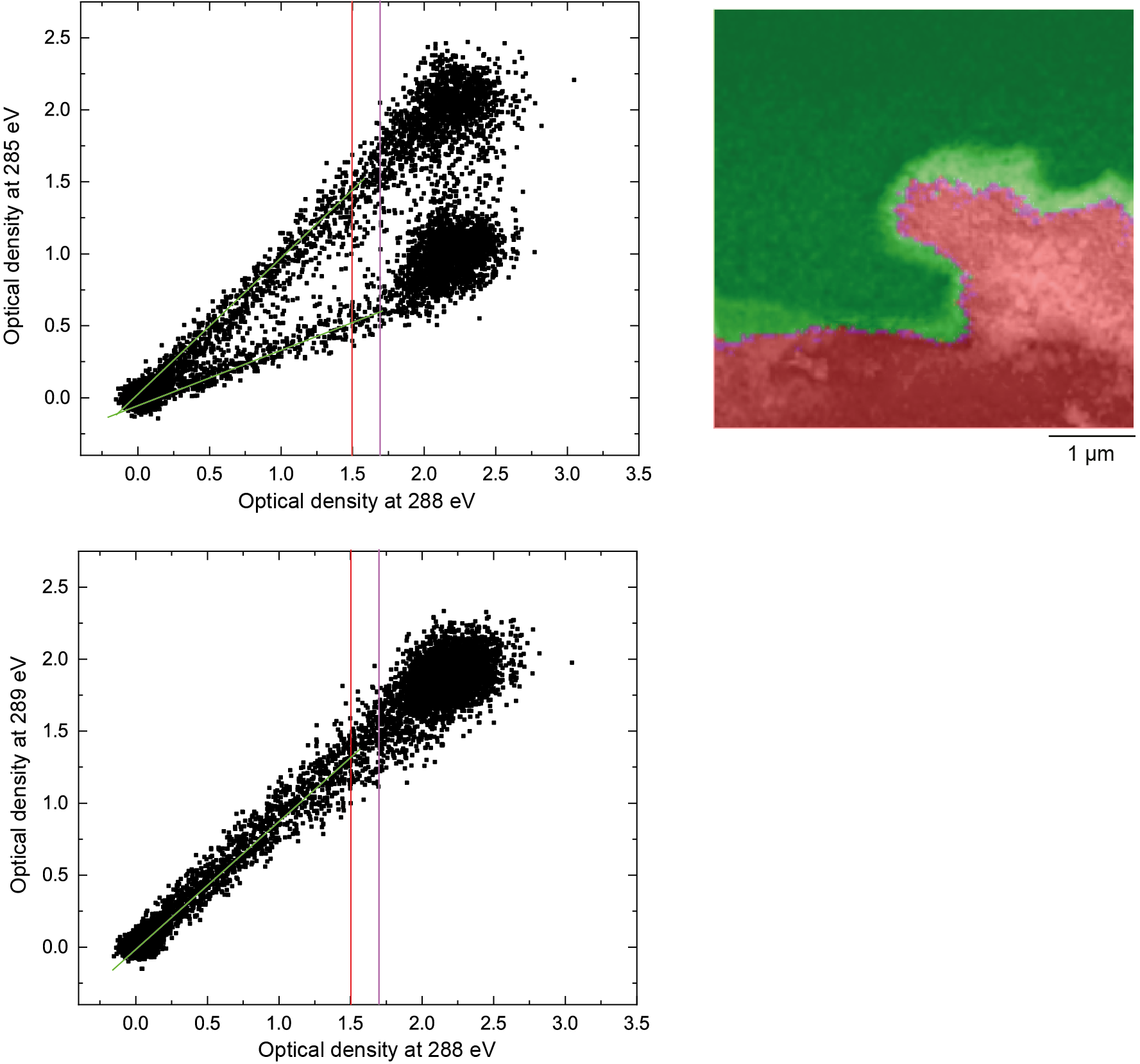


D

**SI Figure 3. Optical density plots showing the limit at which signal saturation is affecting relative intensities of absorption peaks in NEXAFS spectra.** Optical density at amide/carbonyl (288 eV) peak is plotted against either aromatic (285 eV) and/or O-alkyl (289 eV) peak in four selected NEXAFS spectral stacks where signal saturation effects were prominent: A – *P. involutus* no-mineral control sample (Figure 2A in the manuscript), B – *P. subviscida* no-mineral control sample (Figure 2C in the manuscript), C – *P. subviscida* with quartz (Figure 3B in the manuscript) and D – *P. involutus* with quartz (Figure 5B in the manuscript). From the plots, the saturation effect appears significant above optical density values 1.5 - 1.7. On the right, the areas in red and green overlayed on top of corresponding grey scale chemical maps show the areas where the optical density is above and below saturation limit (1.5). Magenta marks the areas where optical density is in the range of 1.5 - 1.7.


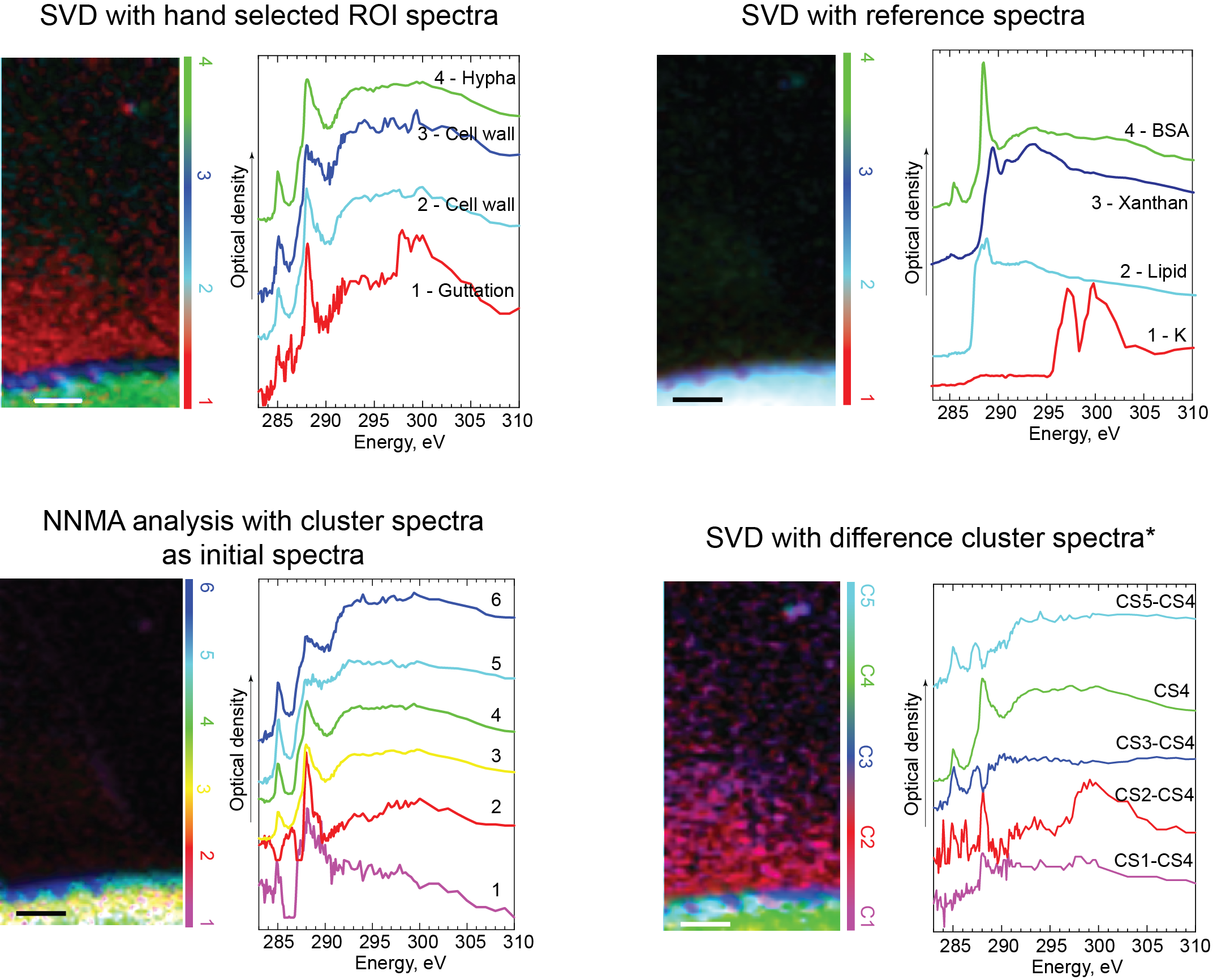


**SI Figure 4.** **Example of different analysis approaches that were tested in this work** (see for comparison with Figure 4 A in the manuscript). Scale bar = 2 μm. *The difference cluster spectra were obtained by iteratively subtracting the cluster spectrum representing the hypha (CS 4) from the other cluster spectra in order to identify those signatures characteristic to the organic compounds present in the exudate halo and the guttation droplet, and different from the hyphal materials. We used the intensity of the peaks at 285 eV and 288 eV as guides for determining the subtraction factor.


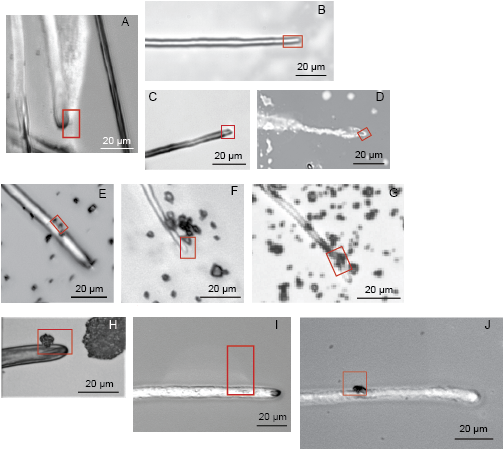


**SI Figure 5.** **Microscopy images of STXM-NEXAFS analyzed fungal hyphae presented in Figures 2-5:** A – *P. involutus* (Figure 2 A), B – *P. subviscida* (Figure 2 C ), C – *G. confluence* (Figure 2 D), D – *R. irregularis* (Figure 2 B), E – *P. involutus* (Figure 3 A), F – *P. subviscida* (Figure 3 B), G – *G. confluence* (Figure 3 C), H – *P. involutus* (Figure 4), I – *P. involutus* (Figure 5 A), J – *P. involutus* (Figure 5 B)


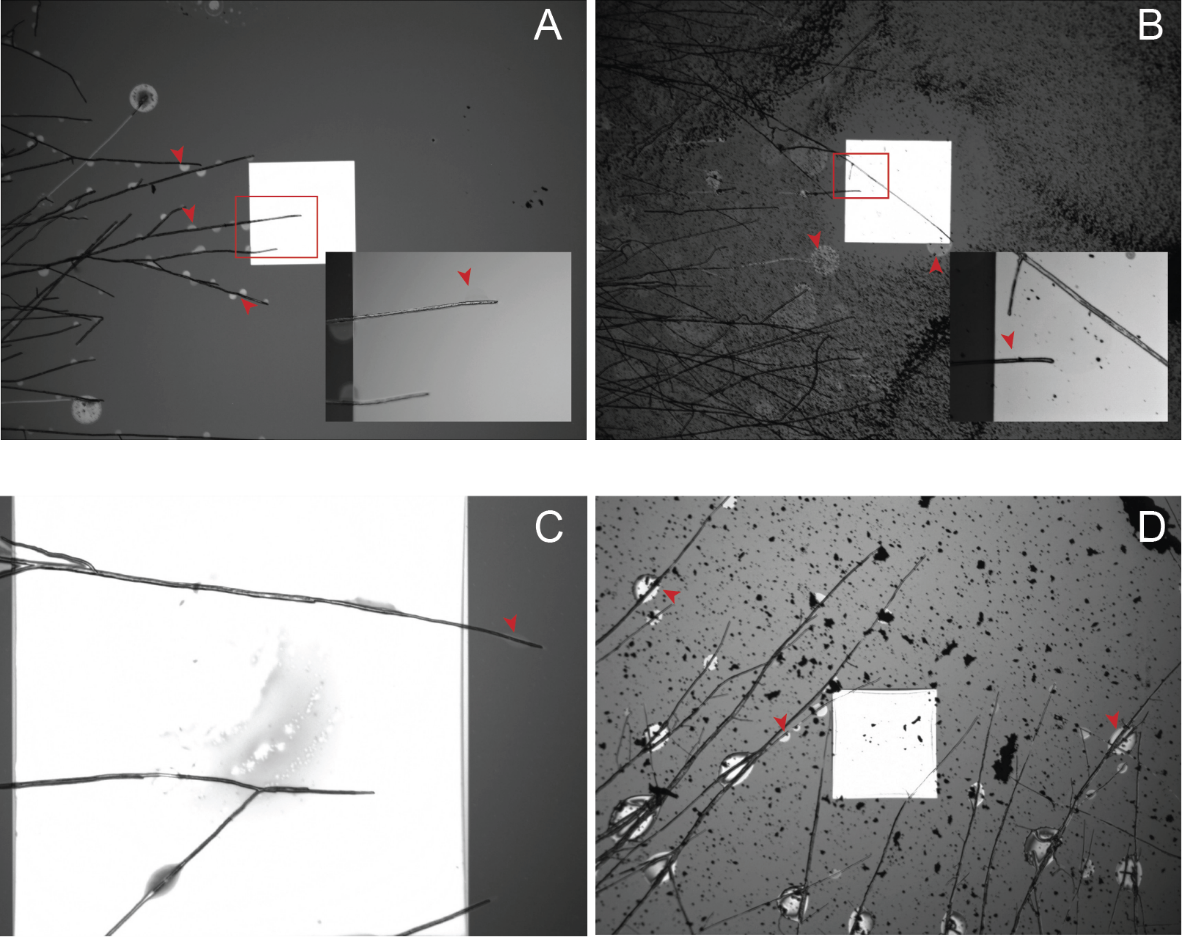


**SI Figure 6.** **Overview stereomicroscopy images silicon nitride membrane windows with fungal hyphae exhibitting guttation:** A – *P. involutus* control sample (shown in Figure 4 A), B – *P. involutus* with quartz particles sample (shown in Figure 4 B), C – *P. subviscida* control sample, D – *P. subviscida* with goethite particles sample


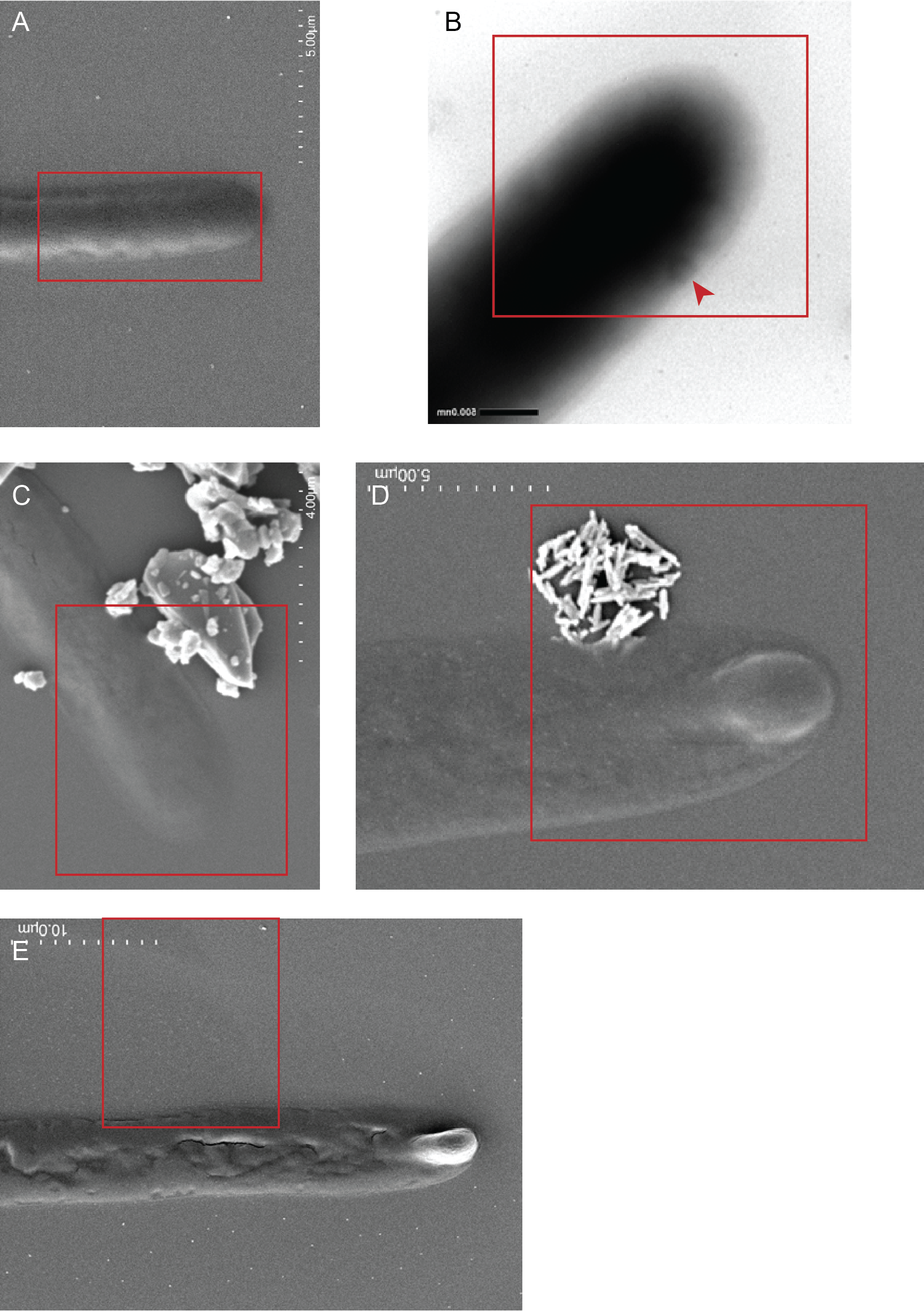


**SI Figure 7.** **Electron microscopy images of selected hyphae that were analyzed by STXM-NEXAFS:** A – SEM image of the analyzed hypha in the *P. subviscida* control sample (refer to Figure 1); B – TEM image the analyzed hypha in the G. confluence control sample (refer to Figure 1), the arrow marks the small circular structure that is also identified in the false-colour pseudo-thickness image in Figure 1; C - SEM image of the analyzed hypha in the *P. subviscida* and quartz particles sample (refer to Figure 2); D - SEM image of the analyzed hypha in the *P. involutus* and goethite particles sample (refer to Figure 3); E - SEM image of the analyzed hypha in the *P. involutus* control sample (refer to Figure 4)


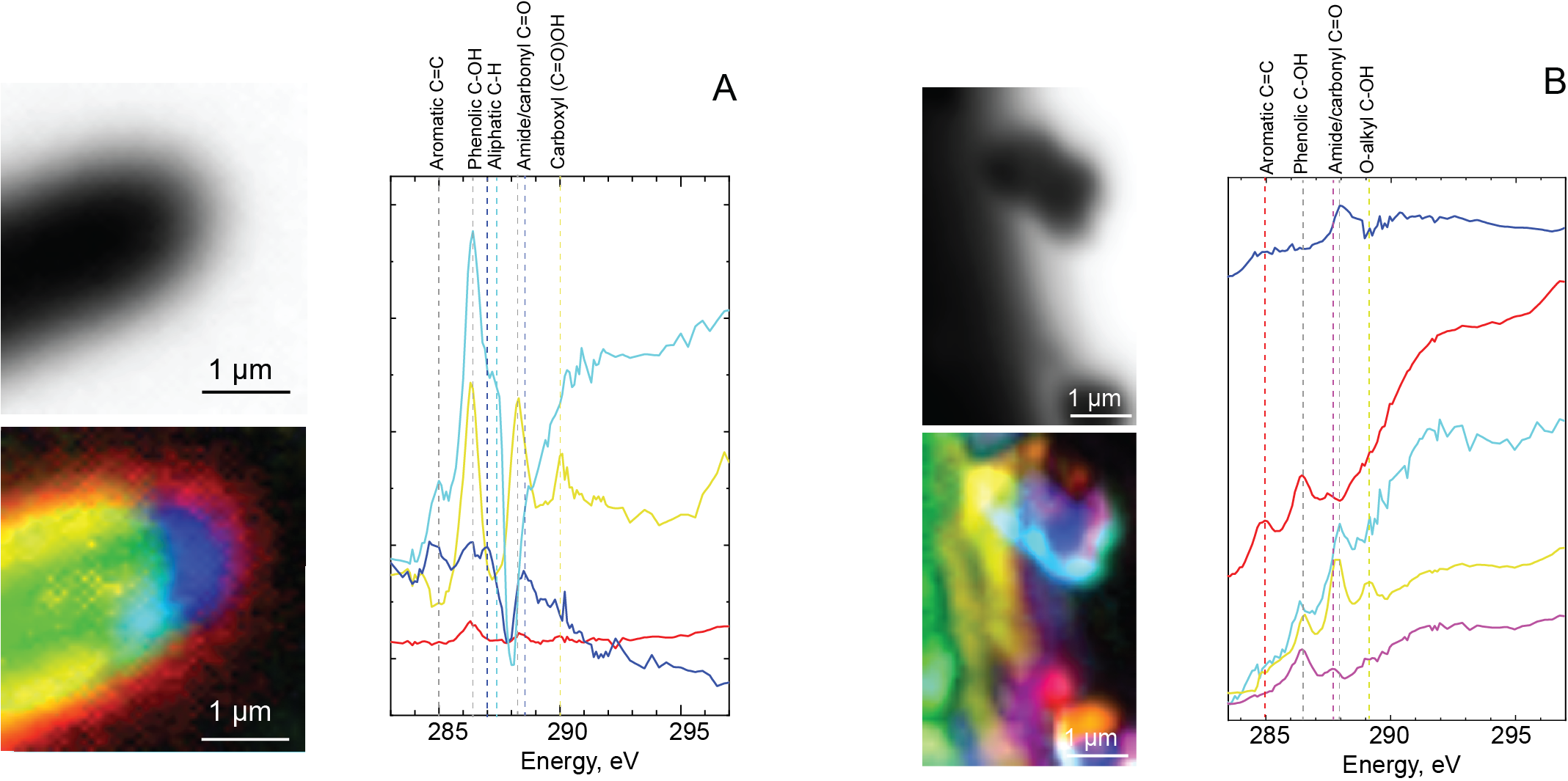


**SI Figure 8.** Difference spectra for A - *G. confluence* control sample and B – *P. involutus* and quartz particles sample.

**
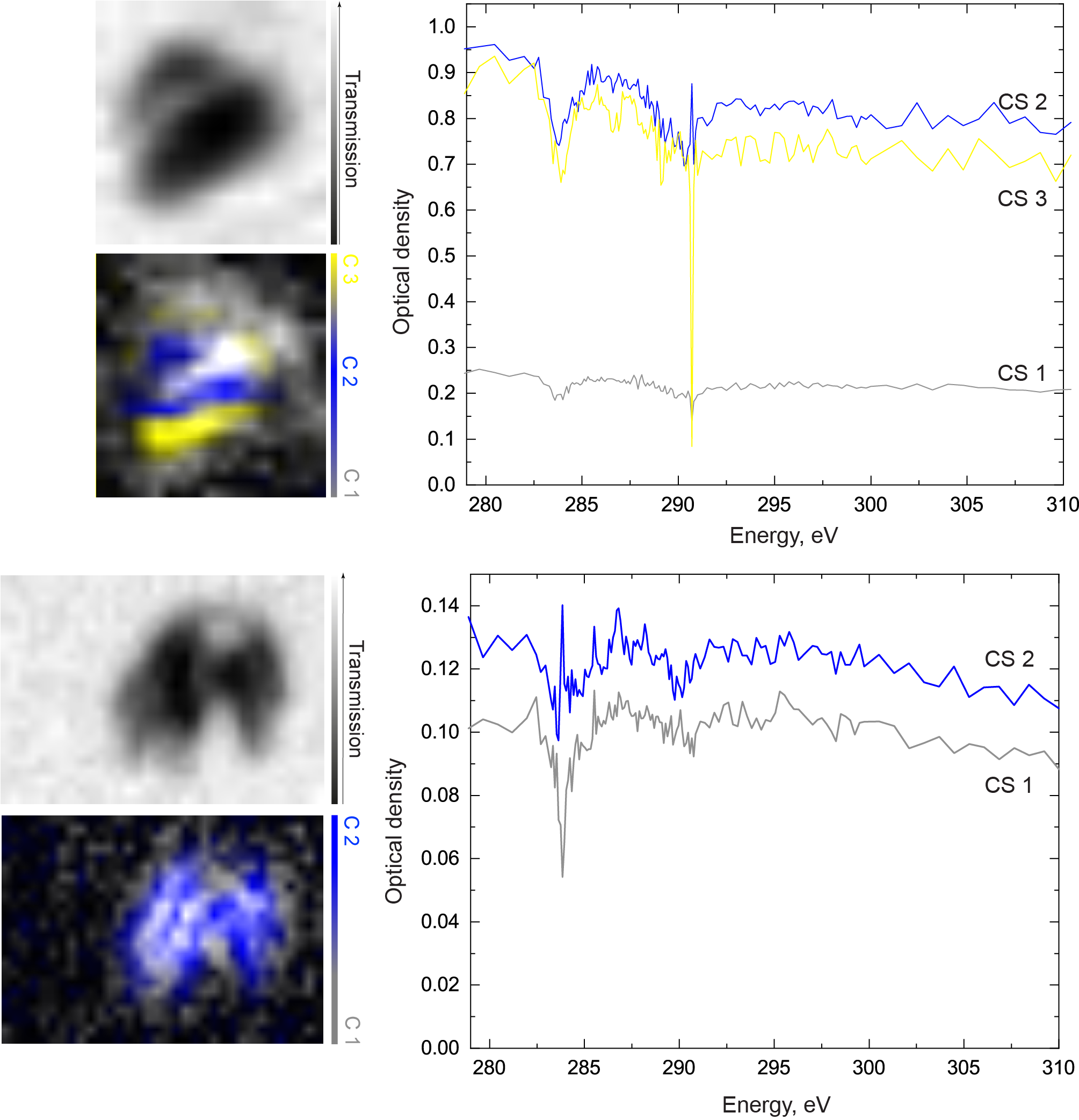
**

A

B

**SI Figure 9.** **Post-edge transmission image (top-left), chemical map (bottom-left) and corresponding cluster spectra (right) of quartz (A) and goethite (B) mineral particles placed far away from the growing hyphae on SiN_x_ membrane windows.** The cluster spectra (the clustering caused by different overall absorbance level) do not contain signatures of C(K) absorption indicating there is no organics deposited on the surface of the particles.

**
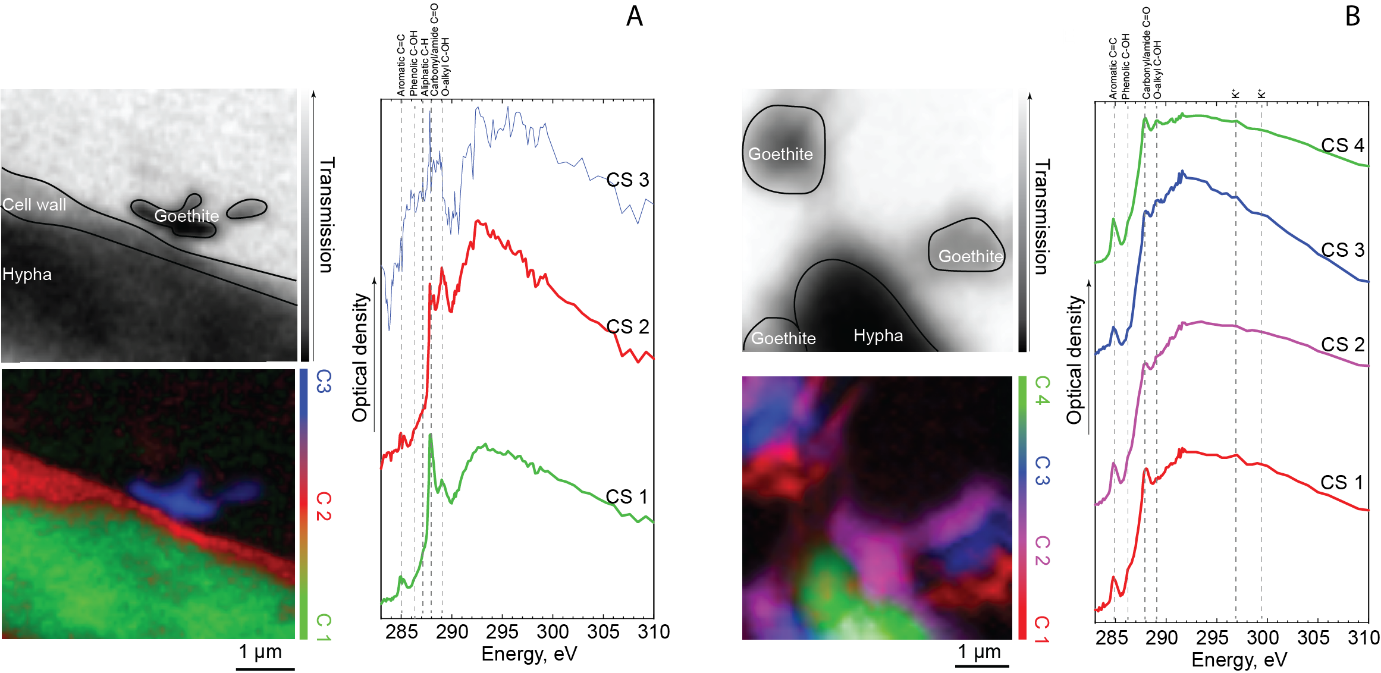
**

**SI Figure 10.** **Post-edge transmission image (top-left), chemical map (bottom-left) and corresponding cluster spectra (right) of *P. subviscida* (A) *and G. confluence* (B) hyphae in contact with goethite particles:** In the chemical map, green color represents the hypha, red – the exudates and blue – the mineral particles.

**SI Table 1.** Cluster spectra peak fitting results. The fitting was performed using Athena software (Ravel, B., and M. Newville. “ATHENA, ARTEMIS, HEPHAESTUS: Data Analysis for X-Ray Absorption Spectroscopy Using IFEFFIT.” Journal of Synchrotron Radiation 12, no. 4 (2005): 537–41.). The arctangent function was loosely set at 290 eV, with the exact position and width allowed to vary. For the peaks, Gaussian function was fitted, with peak positions and widths allowed to vary slightly, but taking care that the width would not exceed 1 eV (typically less). If possible, all of these parameters were fixed for cluster spectra from the same spectral stack, with only edge and peak intensities allowed to vary without constrains. Saturated spectra were omitted from the fitting analysis.

| Sample | Cluster spectrum | Height/Area (OD) | Peak position | Sigma | Step | | | Peak ratio |  |
| --- | --- | --- | --- | --- | --- | --- | --- | --- | --- |
|  |  |  |  |  | Height | Position (E0) | Width |  |  |
|  |  |  |  |  |  |  |  | **Carboxyl/Amide** | **Aromatic/Amide** |
| *P. involutus* no mineral control (Figure 2A) | CS1 | 0.25 | 284.97 | 0.39 | 0.86 | 290.03 | 0.46 |  | 0.28 |
|  |  | 0.42 | 286.67 | 0.6 |  |  |  |  |  |
|  |  | 0.88 | 287.96 | 0.51 |  |  |  |  |  |
|  |  | 0.55 | 289.02 | 0.48 |  |  |  |  |  |
|  |  | 0.31 | 289.82 | 0.48 |  |  |  | 0.35 |  |
|  | CS2 | 0.17 | 284.97 | 0.39 | 0.75 | 290.03 | 0.46 |  | 0.26 |
|  |  | 0.36 | 286.67 | 0.6 |  |  |  |  |  |
|  |  | 0.66 | 287.96 | 0.43 |  |  |  |  |  |
|  |  | 0.46 | 289.02 | 0.41 |  |  |  |  |  |
|  |  | 0.35 | 289.82 | 0.41 |  |  |  | 0.53 |  |
|  | CS3 | 0.26 | 284.97 | 0.36 | 0.94 | 290.08 | 0.46 |  | 0.2 |
|  |  | 0.35 | 286.67 | 0.57 |  |  |  |  |  |
|  |  | 1.13 | 287.96 | 0.59 |  |  |  |  |  |
|  |  | 0.5 | 289.12 | 0.59 |  |  |  |  |  |
|  |  | 0.35 | 289.82 | 0.47 |  |  |  | 0.31 |  |
|  |  |  |  |  |  |  |  |  |  |
|  |  |  |  |  |  |  |  | **Imidazole/Amide** |  |
| *R. irregularis* exudate print (Figure 2B) | CS1 | 0.68 | 286.39 | 0.41 | 1.28 | 289 | 0.5 | 0.45 |  |
|  |  | 1.52 | 288.28 | 0.55 |  |  |  |  |  |
|  | CS2 | 0.59 | 286.39 | 0.34 | 1.44 | 289 | 0.5 | 0.34 |  |
|  |  | 1.72 | 288.28 | 0.53 |  |  |  |  |  |
|  | CS3 | 0.67 | 286.39 | 0.36 | 1.28 | 289 | 0.5 | 0.47 |  |
|  |  | 1.43 | 288.28 | 0.58 |  |  |  |  |  |
|  |  |  |  |  |  |  |  |  |  |
|  |  |  |  |  |  |  |  |  |  |
| *P. subviscida* no mineral control (Figure 2C) | CS1 | 0.24 | 285.14 | 0.31 | 1.16 | 290.33 | 0.57 |  |  |
|  |  | 1.51 | 288.02 | 0.68 |  |  |  |  |  |
|  |  | 0.92 | 289.43 | 0.64 |  |  |  |  |  |
|  | CS2 | 0.2 | 285.14 | 0.46 | 1.57 | 290.33 | 0.57 |  |  |
|  |  | 1.26 | 288.02 | 0.8 |  |  |  |  |  |
|  |  | 1.42 | 289.43 | 0.65 |  |  |  |  |  |
|  | CS3 | 0.26 | 285.14 | 0.3 | 1.59 | 290.33 | 0.57 |  |  |
|  |  | 1.76 | 288.02 | 0.6 |  |  |  |  |  |
|  |  | 1.4 | 289.43 | 0.64 |  |  |  |  |  |
|  |  |  |  |  |  |  |  | **Phenolic/Amide** |  |
| *G. confluence* no mineral control (Figure 2D) | CS1 | 0.13 | 284.95 | 0.32 | 0.88 | 288.93 | 0.39 |  |  |
|  |  | 0.26 | 286.37 | 0.4 |  |  |  | 0.22 |  |
|  |  | 1.2 | 288 | 0.66 |  |  |  |  |  |
|  | CS2 | 0.12 | 284.95 | 0.33 | 0.9 | 288.93 | 0.39 |  |  |
|  |  | 0.26 | 286.37 | 0.37 |  |  |  | 0.21 |  |
|  |  | 1.22 | 288 | 0.66 |  |  |  |  |  |
|  | CS3 | 0.13 | 284.95 | 0.32 | 0.89 | 288.93 | 0.39 |  |  |
|  |  | 0.22 | 286.37 | 0.41 |  |  |  | 0.18 |  |
|  |  | 1.22 | 288 | 0.67 |  |  |  |  |  |
|  | CS4 | 0.15 | 284.95 | 0.32 | 0.93 | 288.93 | 0.39 |  |  |
|  |  | 0.24 | 286.37 | 0.41 |  |  |  | 0.18 |  |
|  |  | 1.36 | 288 | 0.69 |  |  |  |  |  |
|  | CS5 | 0.13 | 284.95 | 0.31 | 0.87 | 288.93 | 0.39 |  |  |
|  |  | 0.29 | 286.37 | 0.44 |  |  |  | 0.26 |  |
|  |  | 1.13 | 288 | 0.68 |  |  |  |  |  |
|  |  |  |  |  |  |  |  |  |  |
|  |  |  |  |  |  |  |  | **Phenolic/Amide** | **Aromatic/Amide** |
| *P. involutus* with quartz (Figure 3A) | CS1 | 0.14 | 285 | 0.36 | 0.8 | 289.39 | 0.48 |  | 0.23 |
|  |  | 0.33 | 286.61 | 0.54 |  |  |  | 0.55 |  |
|  |  | 0.6 | 287.96 | 0.51 |  |  |  |  |  |
|  |  | 0.26 | 289.02 | 0.38 |  |  |  |  |  |
|  |  |  |  |  |  |  |  |  |  |
|  | CS2 | 0.18 | 285 | 0.35 | 0.95 | 289.39 | 0.48 |  | 0.22 |
|  |  | 0.44 | 286.61 | 0.58 |  |  |  | 0.53 |  |
|  |  | 0.83 | 287.96 | 0.55 |  |  |  |  |  |
|  |  | 0.33 | 289.02 | 0.39 |  |  |  |  |  |
|  | CS3 | 0.16 | 285 | 0.34 | 0.9 | 289.39 | 0.48 |  | 0.21 |
|  |  | 0.43 | 286.61 | 0.59 |  |  |  | 0.57 |  |
|  |  | 0.75 | 287.96 | 0.53 |  |  |  |  |  |
|  |  | 0.32 | 289.02 | 0.39 |  |  |  |  |  |
|  | CS4 | 0.14 | 285 | 0.36 | 0.81 | 289.39 | 0.48 |  | 0.22 |
|  |  | 0.29 | 286.61 | 0.54 |  |  |  | 0.46 |  |
|  |  | 0.63 | 287.96 | 0.53 |  |  |  |  |  |
|  |  | 0.26 | 289.02 | 0.38 |  |  |  |  |  |
|  | CS5 | 0.25 | 285 | 0.43 | 0.85 | 289.39 | 0.48 |  | 0.33 |
|  |  | 0.38 | 286.61 | 0.54 |  |  |  | 0.51 |  |
|  |  | 0.75 | 287.96 | 0.53 |  |  |  |  |  |
|  |  | 0.33 | 289.02 | 0.44 |  |  |  |  |  |
|  | CS6 | 0.18 | 285 | 0.37 | 0.84 | 289.39 | 0.48 |  | 0.25 |
|  |  | 0.34 | 286.61 | 0.54 |  |  |  | 0.47 |  |
|  |  | 0.72 | 287.96 | 0.51 |  |  |  |  |  |
|  |  | 0.31 | 289.02 | 0.4 |  |  |  |  |  |
|  | CS7 | 0.16 | 285 | 0.37 | 0.79 | 289.39 | 0.48 |  | 0.27 |
|  |  | 0.4 | 286.61 | 0.6 |  |  |  | 0.68 |  |
|  |  | 0.59 | 287.96 | 0.54 |  |  |  |  |  |
|  |  | 0.29 | 289.02 | 0.42 |  |  |  |  |  |
|  |  |  |  |  |  |  |  |  |  |
| *P. subviscida* with quartz (Figure 3B) | CS1* | 1.59 | 288.13 | 1.48 | 1.83 | 290.75 | 0.41 |  |  |
|  |  | 1.59 | 289.25 | 0.77 |  |  |  |  |  |
|  | CS2 | 0.14 | 285.06 | 0.25 | 1.52 | 289.91 | 0.81 |  |  |
|  |  | 1.67 | 288.13 | 0.55 |  |  |  |  |  |
|  |  | 0.46 | 289.25 | 0.37 |  |  |  |  |  |
|  | CS3 | 0.07 | 285.06 | 0.21 | 1.88 | 289.91 | 0.81 |  |  |
|  |  | 1.3 | 288.13 | 0.52 |  |  |  |  |  |
|  |  | 0.76 | 289.25 | 0.39 |  |  |  |  |  |
|  |  |  |  |  |  |  |  |  |  |
|  |  |  |  |  |  |  |  | **Phenolic/Amide** | **Aromatic/Amide** |
| *G. confluence* with quartz (Figure 3C) | CS1 | 0.17 | 284.9 | 0.32 | 0.87 | 289.73 | 0.3 |  | 0.17 |
|  |  | 0.42 | 286.6 | 0.62 |  |  |  | 0.42 |  |
|  |  | 1 | 287.97 | 0.56 |  |  |  |  |  |
|  |  | 0.69 | 289.2 | 0.48 |  |  |  |  |  |
|  | CS2 | 0.18 | 284.9 | 0.35 | 0.89 | 289.73 | 0.3 |  | 0.18 |
|  |  | 0.49 | 286.6 | 0.62 |  |  |  | 0.49 |  |
|  |  | 1 | 287.97 | 0.57 |  |  |  |  |  |
|  |  | 0.75 | 289.2 | 0.51 |  |  |  |  |  |
|  | CS3** |  |  |  |  |  |  |  |  |
|  |  |  |  |  |  |  |  |  |  |
| *P. involutus* with goethite (Figure 4) | CS1** |  |  |  |  |  |  |  |  |
|  | CS2 | 0.15 | 285.06 | 0.29 | 0.99 | 290.08 | 0.62 |  |  |
|  |  | 0.22 | 287.16 | 0.34 |  |  |  |  |  |
|  |  | 0.56 | 288.02 | 0.32 |  |  |  |  |  |
|  |  | 1.03 | 288.98 | 0.7 |  |  |  |  |  |
|  | CS3** |  |  |  |  |  |  |  |  |
|  |  |  |  |  |  |  |  |  |  |
| *P. involutus* no mineral control (Figure 5A) | CS1 | 0.09 | 285.2 | 0.21 | 1.39 | 290.13 | 0.92 |  |  |
|  |  | 1.33 | 288.19 | 0.55 |  |  |  |  |  |
|  |  | 0.41 | 289.47 | 0.42 |  |  |  |  |  |
|  | CS2 | 0.28 | 285.2 | 0.25 | 0.14 | 290.13 | 0.92 |  |  |
|  |  | 1.62 | 288.19 | 0.74 |  |  |  |  |  |
|  |  | 0.09 | 289.47 | 0.74 |  |  |  |  |  |
|  | CS3* | 0.35 | 285.2 | 0.37 | 1.03 | 290.13 | 0.55 |  |  |
|  |  | 0.68 | 287.37 | 0.92 |  |  |  |  |  |
|  |  | 0.79 | 288.19 | 0.64 |  |  |  |  |  |
|  |  | 0.78 | 289.47 | 0.63 |  |  |  |  |  |
|  | CS4 | 0.17 | 285.2 | 0.29 | 1.2 | 290.13 | 0.92 |  |  |
|  |  | 1.35 | 288.19 | 0.64 |  |  |  |  |  |
|  |  | 0.29 | 289.47 | 0.4 |  |  |  |  |  |
|  | CS5* | 0.27 | 285.2 | 0.35 | 1.01 | 290.13 | 0.55 |  |  |
|  |  | 0.25 | 286.66 | 0.74 |  |  |  |  |  |
|  |  | 0.06 | 287.14 | 0.21 |  |  |  |  |  |
|  |  | 1.04 | 288.19 | 0.64 |  |  |  |  |  |
|  |  | 0.46 | 289.47 | 0.48 |  |  |  |  |  |
|  |  |  |  |  |  |  |  |  |  |
|  |  |  |  |  |  |  |  |  |  |
| *P. involutus* with quartz (Figure 5B) | CS1** |  |  |  |  |  |  |  |  |
|  | CS2 | 0.17 | 285.09 | 0.19 | 0.88 | 289.98 | 0.95 |  |  |
|  |  | 1.39 | 288.14 | 0.81 |  |  |  |  |  |
|  |  | 0.25 | 289.58 | 0.53 |  |  |  |  |  |
|  | CS5** |  |  |  |  |  |  |  |  |

*edge energy position needed to be relaxed for a good fit

**the cluster spectra were too noisy or negative features indicating saturation (at least in part of the cluster spectrum) to achieve a good fit

**SI Table 2. Fe(II) peak intensities at 708 eV in the intensity-normalized NEXAFS spectra recorded in samples of diferrent soil fungi in contact with goethite minerals. ‘Free’ goethite spectra were recorded in areas without fungal hyphae in the vicinity for reference. Summary of intensity averages and variance as well as results from t-test for goethite at *P. involutus* compared with ‘free’ goethite is shown lower in the table.**

|  | Free' goethite | Goethite at *P. involutus* | Goethite at *P. subviscida* | Goethite at *G. confluence* |
| --- | --- | --- | --- | --- |
| Sample 1 | 0.75 | 0.86 | 0.64 | 0.73 |
| Sample 2 | 0.83 | 0.92 | 0.67 | 0.73 |
| Sample 3 | 0.68 | 0.78 |  |  |
| SUMMARY | | | | |
| *Groups* | *Count* | *Average* | *Variance* |  |
| Free' goethite | 3 | 0.75 | 0.006 |  |
| Goethite at *P. involutus* | 3 | 0.85 | 0.005 |  |
| Goethite at *P. subviscida* | 2 | 0.66 | 0.0005 |  |
| Goethite at *G. confluence* | 2 | 0.73 | 0 |  |
|  |  |  |  |  |
| t-Test: Paired Two Sample for Means | | | | |
|  | Goethite at *P. involutus* | Free' goethite |  |  |
| Mean | 0.85 | 0.75 |  |  |
| Variance | 0.005 | 0.006 |  |  |
| Observations | 3 | 3 |  |  |
| Pearson Correlation | 0.99 |  |  |  |
| Hypothesized Mean Difference | 0 |  |  |  |
| df | 2 |  |  |  |
| t Stat | 17.32 |  |  |  |
| P(T<=t) one-tail | 0.002 |  |  |  |
| t Critical one-tail | 2.92 |  |  |  |
| P(T<=t) two-tail | 0.003 |  |  |  |
| t Critical two-tail | 4.30 |  |  |  |


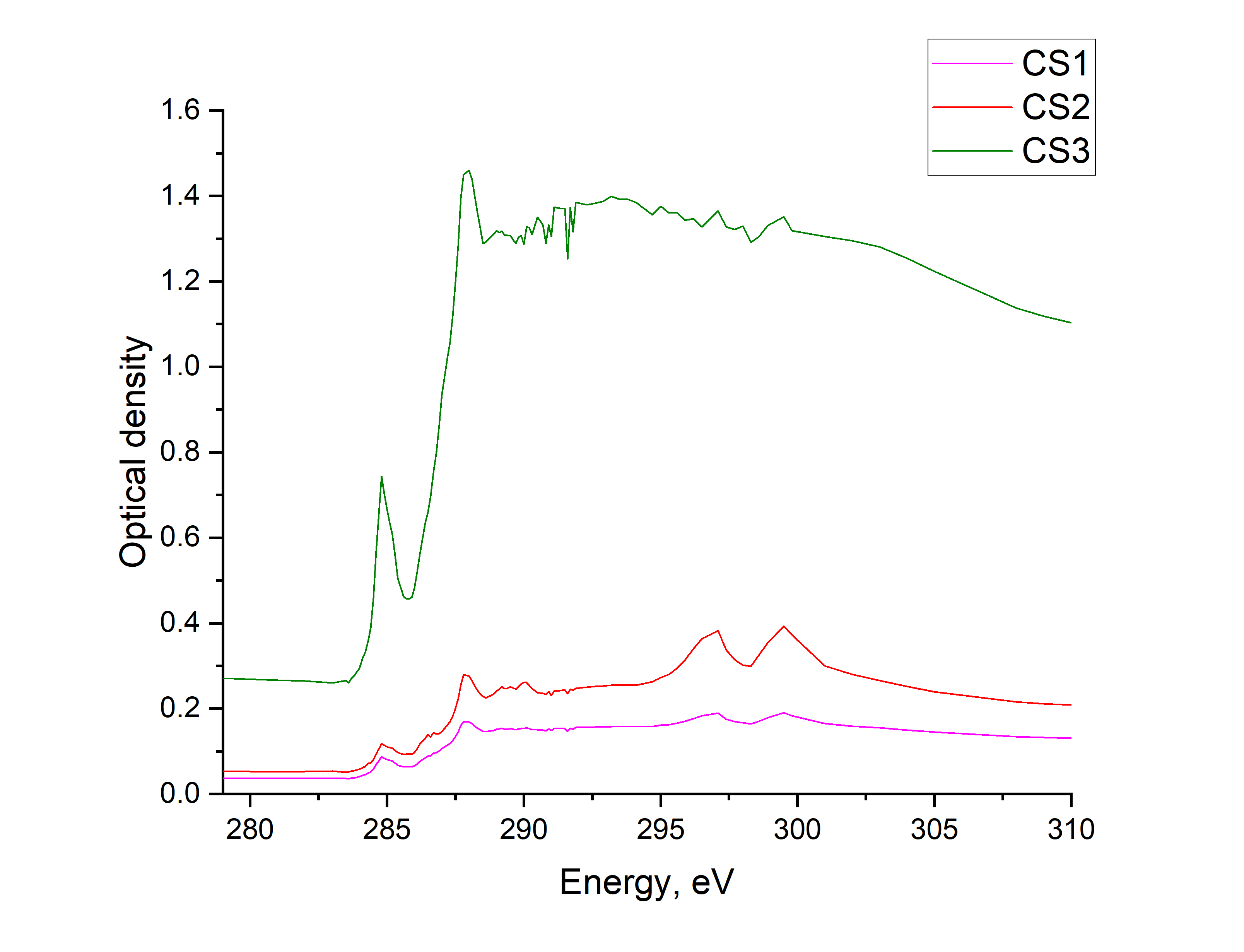


A

**SI Figure 11.** **Spectral graphs showing cluster spectra in the original optical density scale:** A - *P. involutus* no- mineral control sample (Figure 2A), B – *R. irregularis* no-mineral control sample (Figure 2B), C – *P. subviscida* no-mineral control sample (Figure 2C), D – *G. confluence* no-mineral control sample (Figure 2D), E - *P. involutus* with quartz sample (Figure 3A), F – *P. subviscida* with quartz sample (Figure 3B), G – *G. confluence* with quartz sample (Figure 3C), H – *P. involutus* with goethite sample (Figure 4), I – *P. involutus* no-mineral control sample (Figure 5A), J - *P. involutus* with quartz sample (Figure 5B). Continued in subsequent pages.


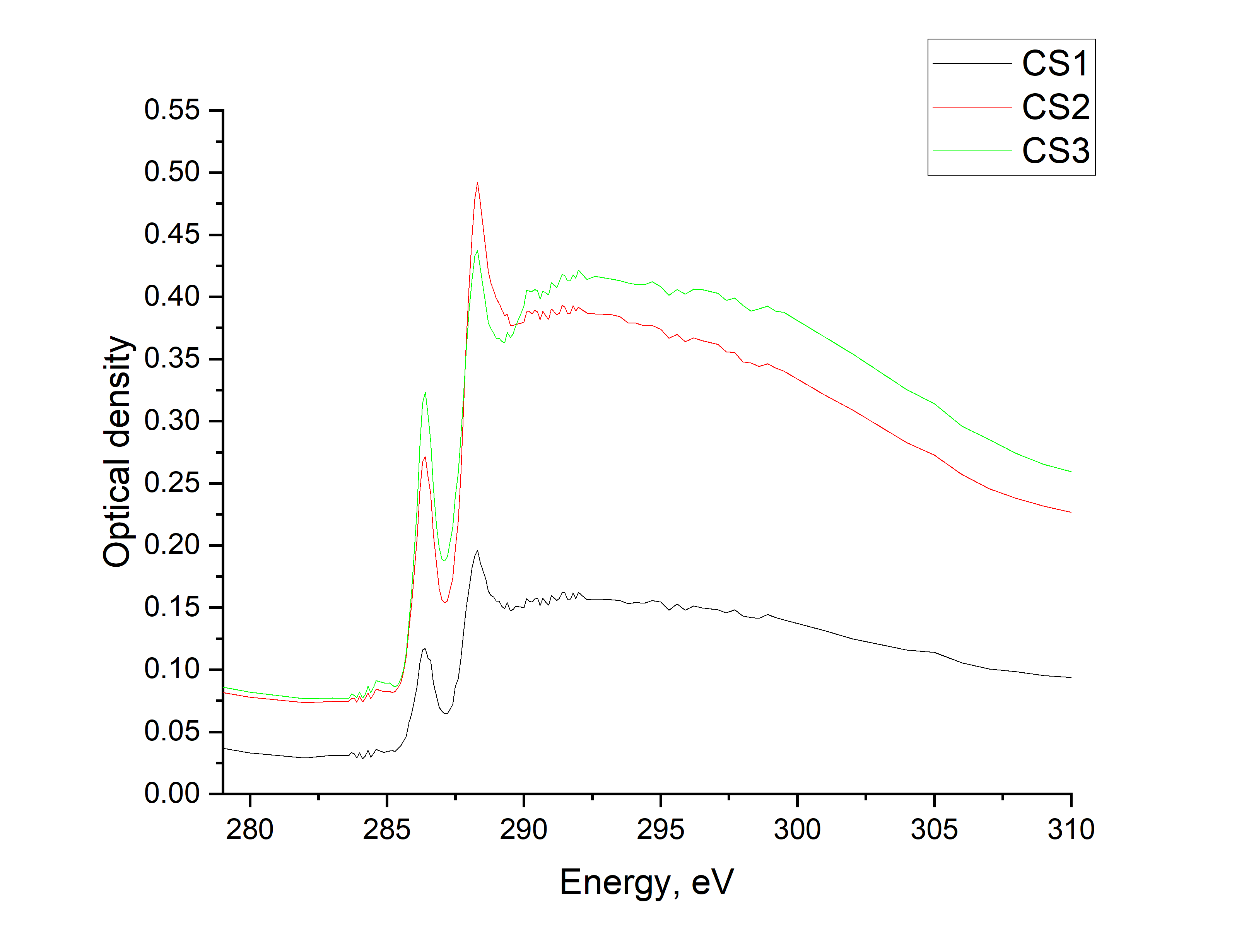

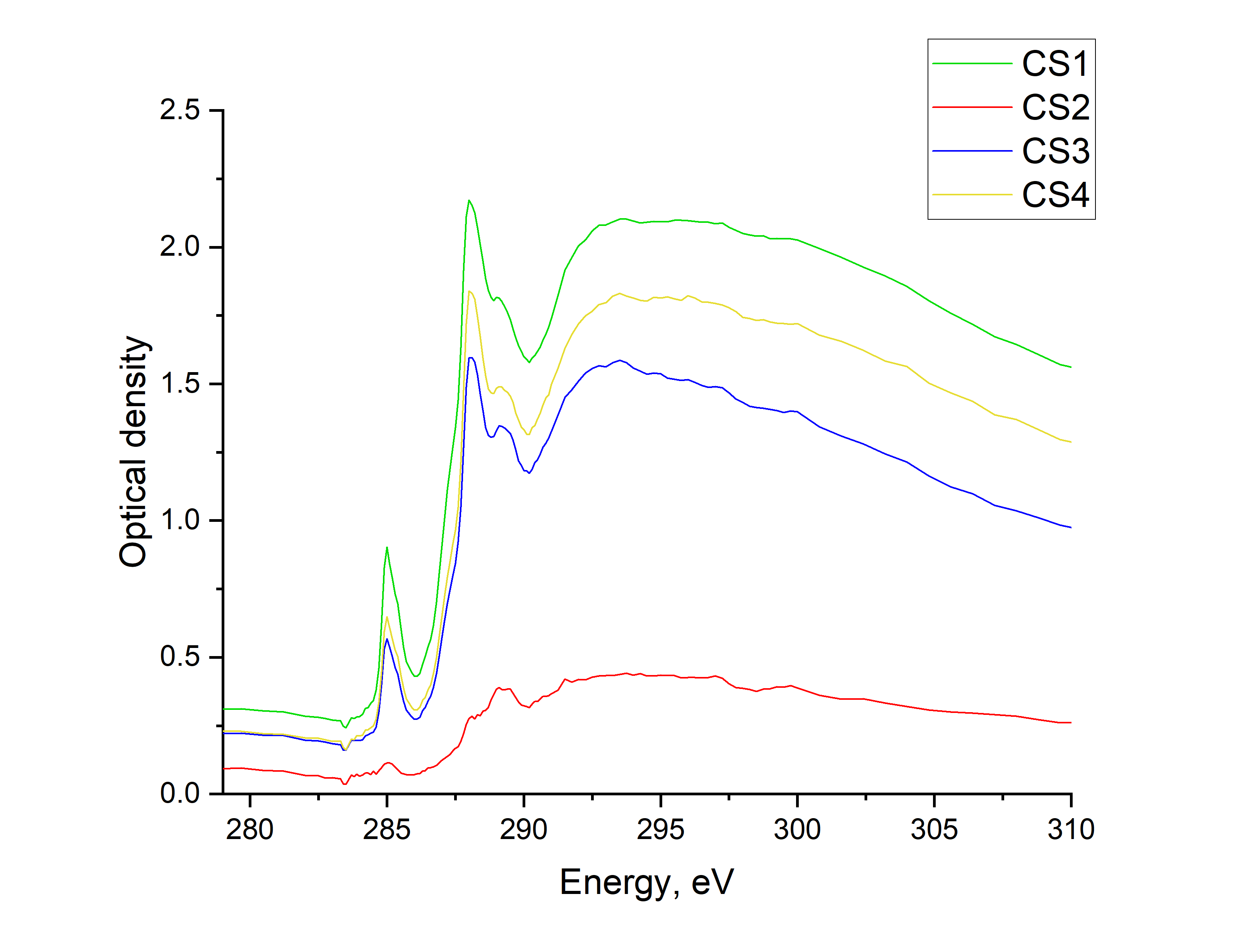

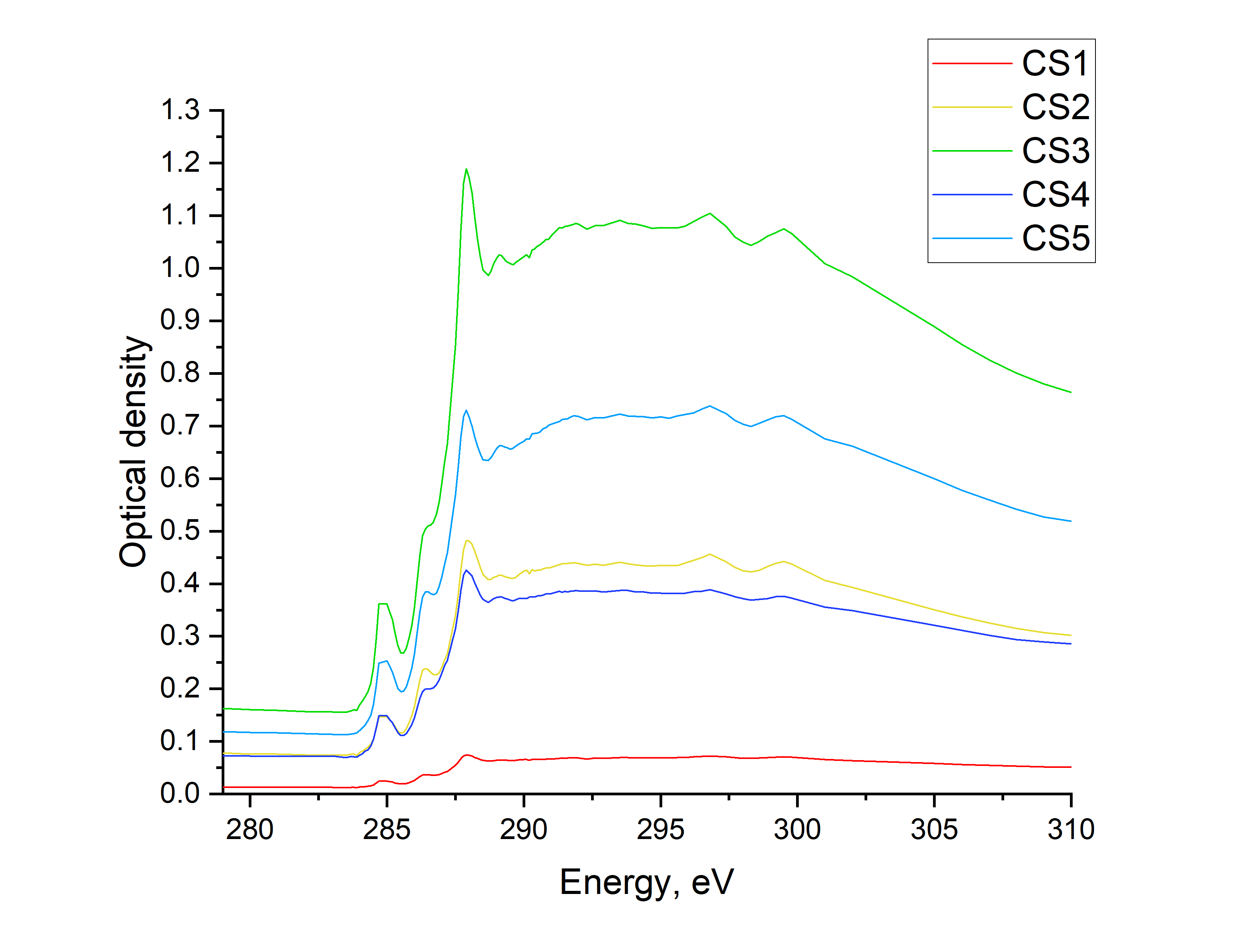

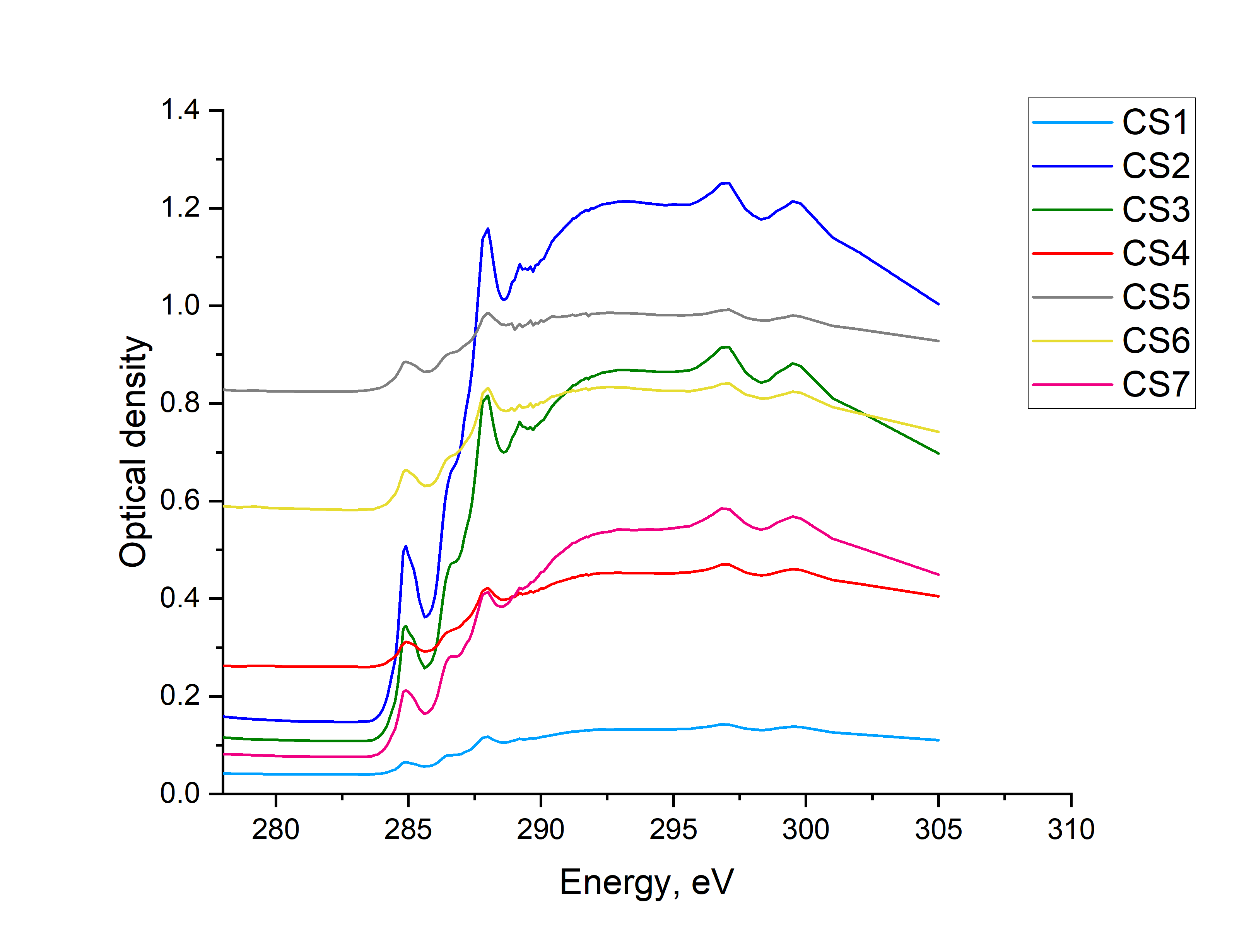

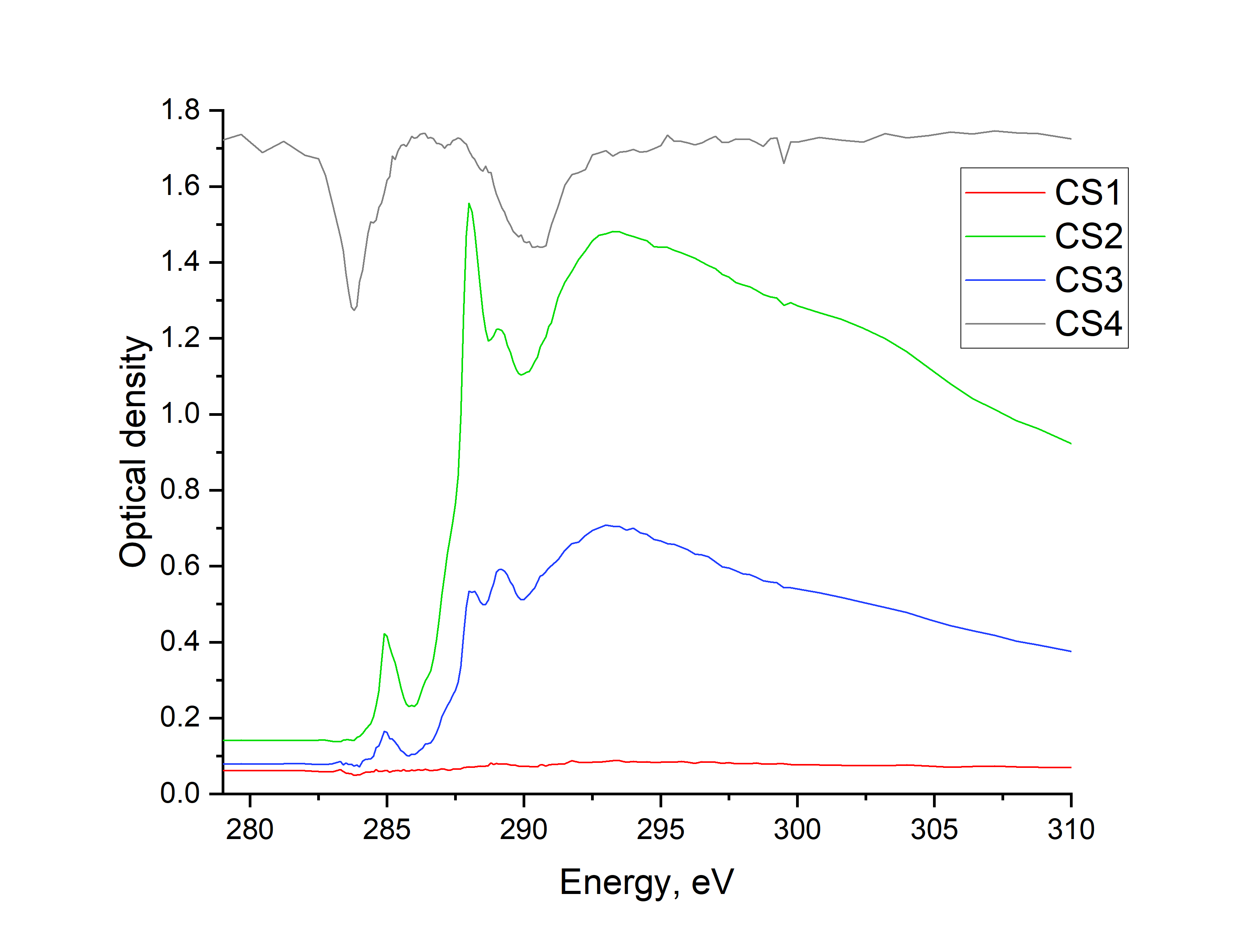

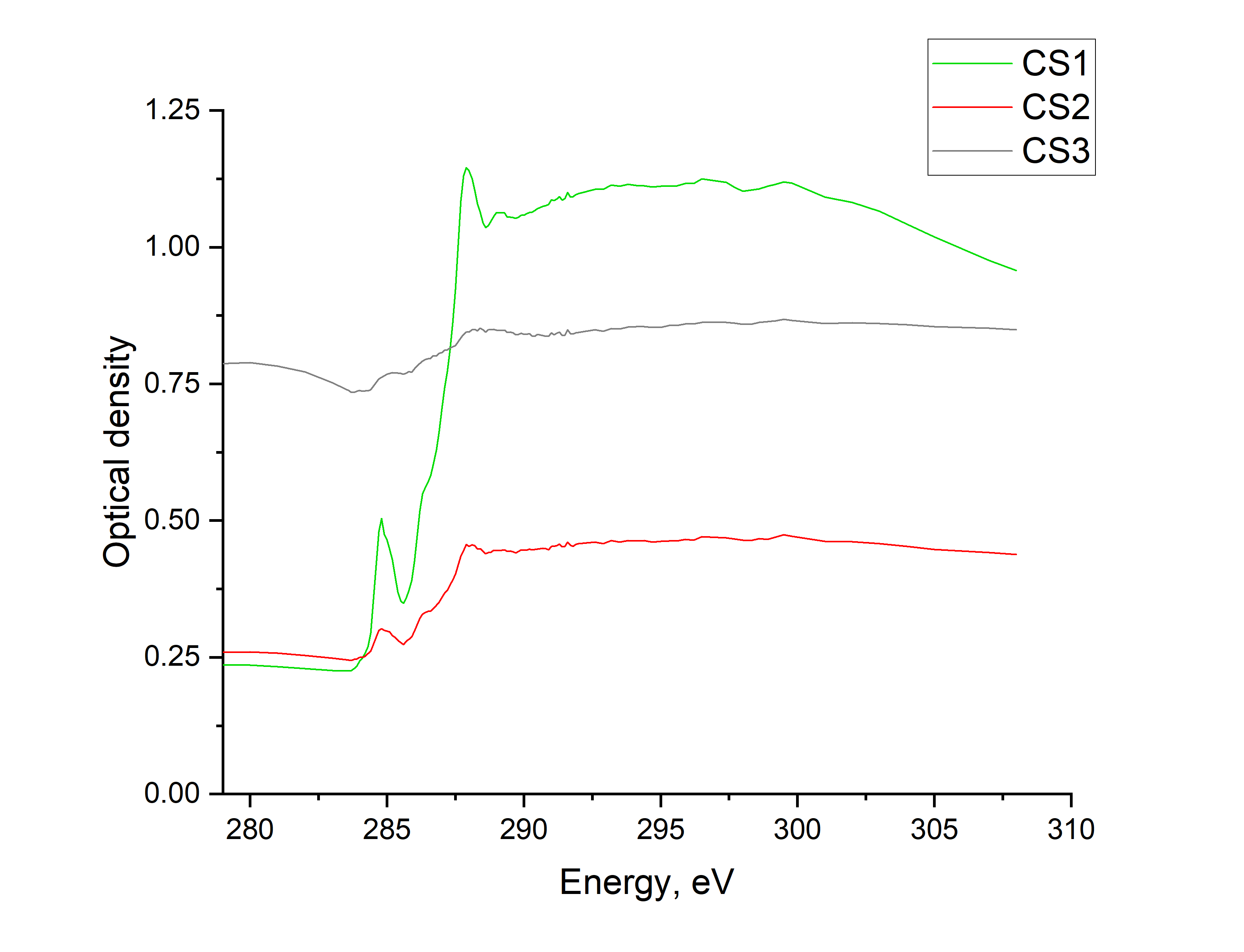

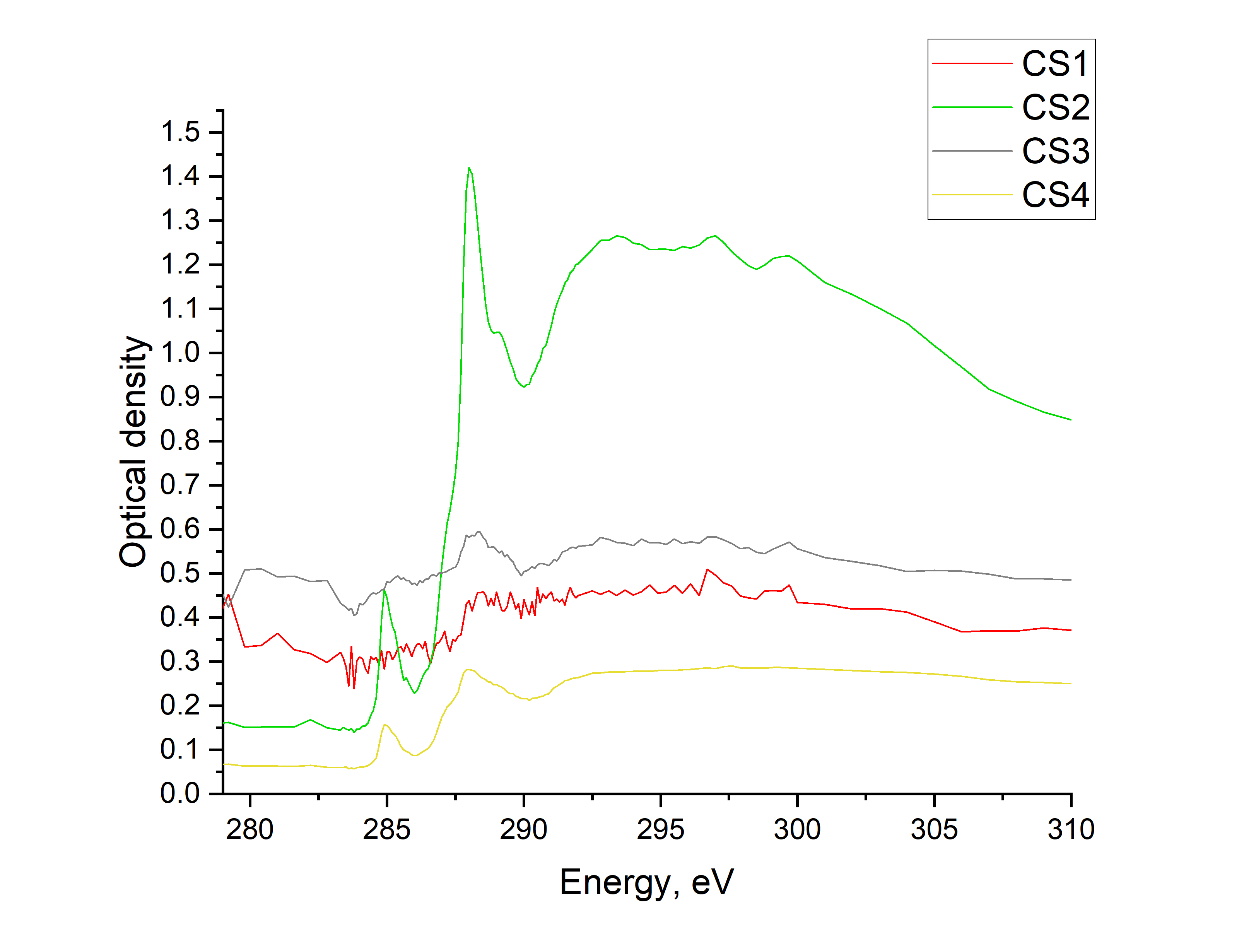

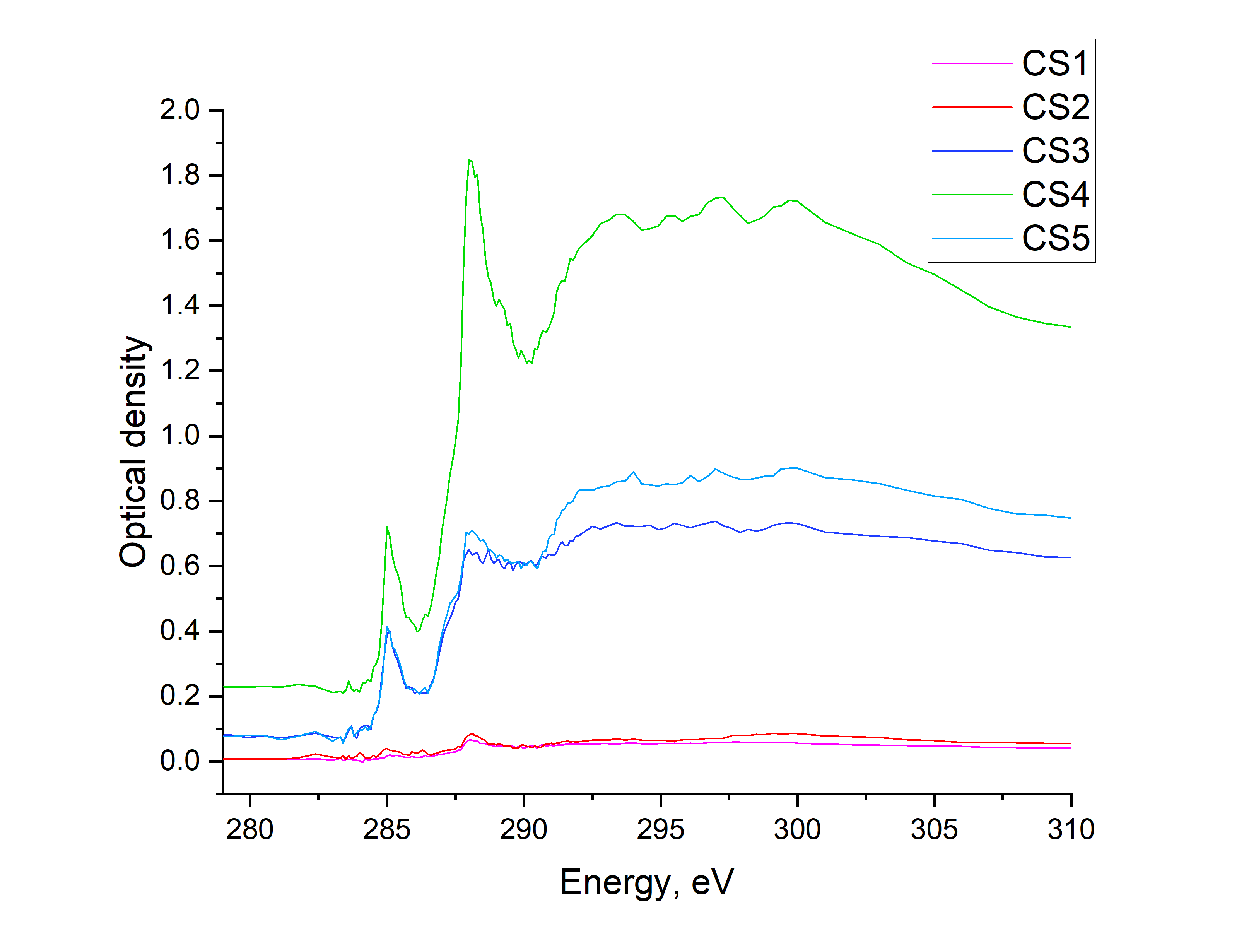


B

C

D

E

F

G

H

I


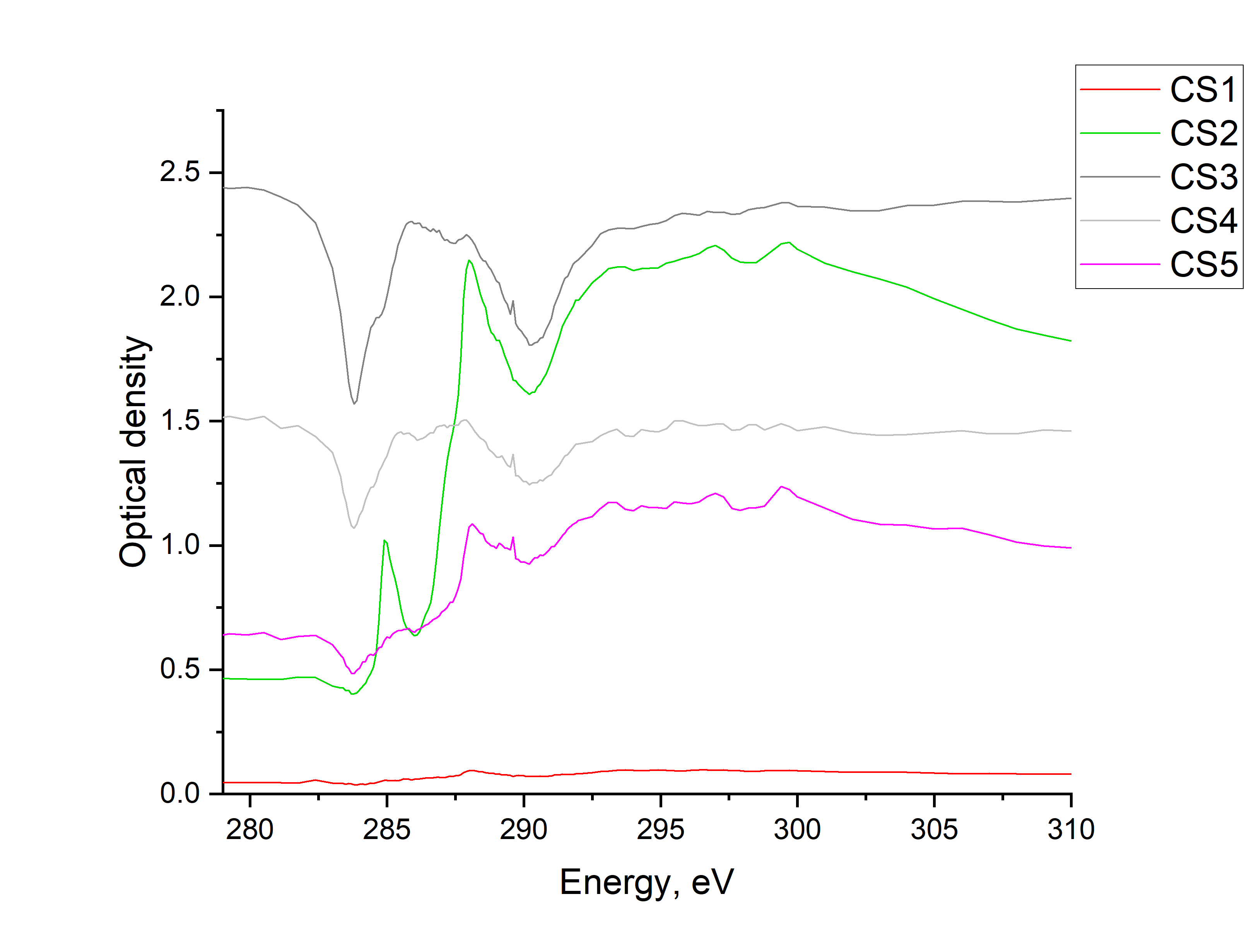


J


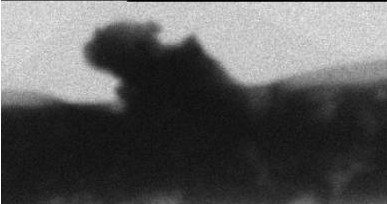

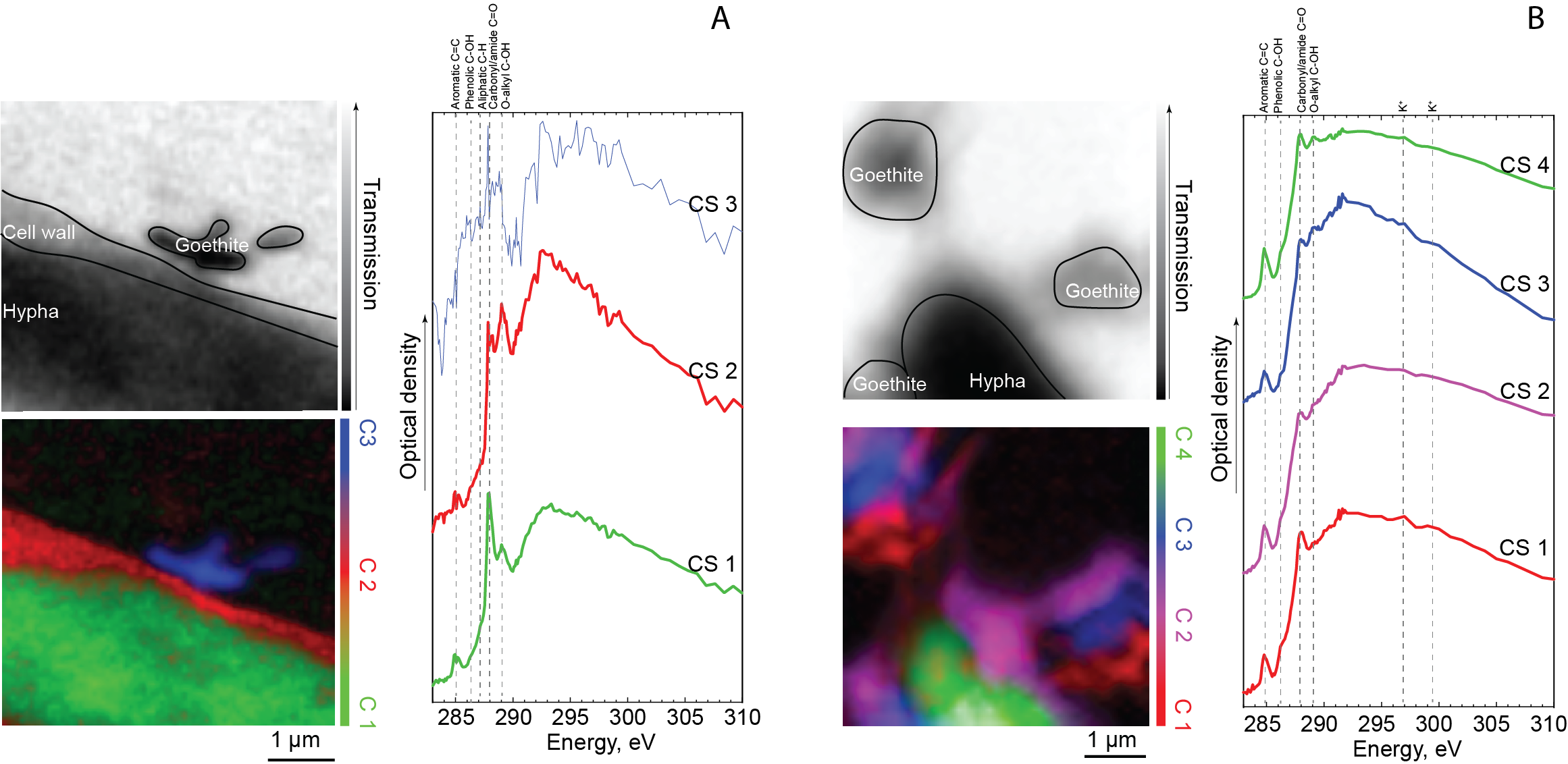


**SI Figure 12.** **Single transmission image of ectomycorrhizal fungus *P. involutus* hypha in contact with quartz particle recorded at 288.2 eV.** In this overview image recorded prior collecting full spectra stack, the guttation droplet is clearly seen to be present around the quartz particle.


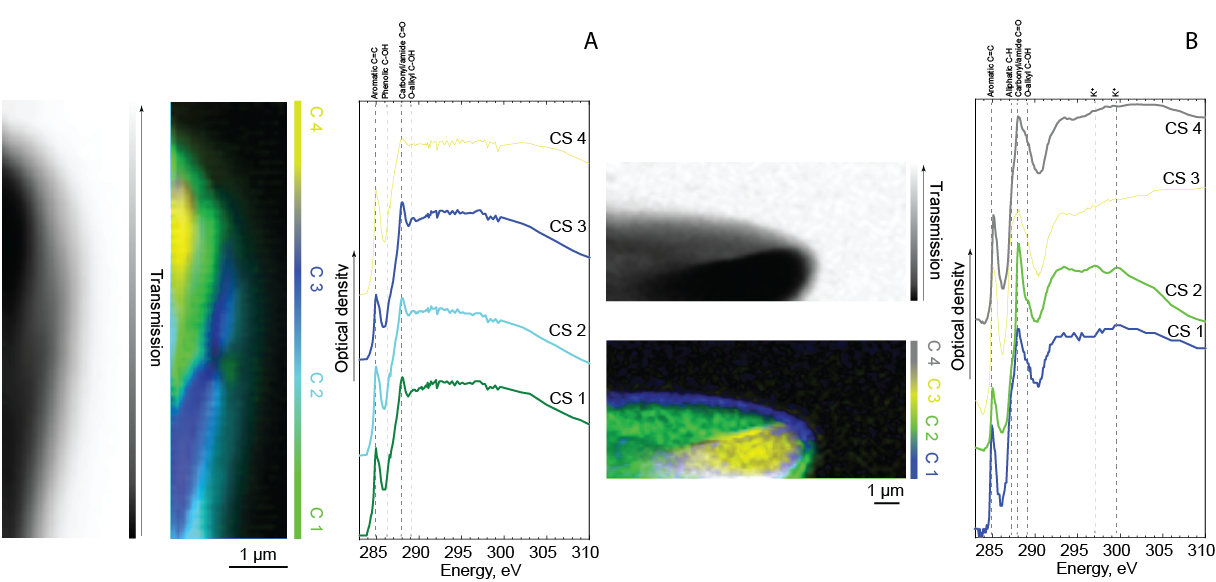


**SI Figure 13.** **Post-edge transmission image (A - left, B - top-left), chemical map (A - middle, B - bottom-left) and corresponding cluster spectra (right) of *P. involutus* hyphae not exhibiting any exudation:** In the chemical map, green/yellow color represents the hypha, blue/cyan – the cell-wall.
